# Cost-effectiveness of Alternative Approaches to Hepatitis C Diagnosis and Treatment Initiation for Treatment-naive People Who Inject Drugs in Australia: A Model-based Economic Evaluation

**DOI:** 10.1093/ofid/ofaf514

**Published:** 2025-08-22

**Authors:** Christopher R Bailie, Nick Scott, Alisa E Pedrana, Margaret E Hellard, Joseph S Doyle

**Affiliations:** Disease Elimination Program, Burnet Institute, Melbourne, Victoria, Australia; Doherty Institute, University of Melbourne, Melbourne, Victoria, Australia; Disease Elimination Program, Burnet Institute, Melbourne, Victoria, Australia; School of Public Health and Preventive Medicine, Monash University, Melbourne, Victoria, Australia; Disease Elimination Program, Burnet Institute, Melbourne, Victoria, Australia; School of Public Health and Preventive Medicine, Monash University, Melbourne, Victoria, Australia; Disease Elimination Program, Burnet Institute, Melbourne, Victoria, Australia; Doherty Institute, University of Melbourne, Melbourne, Victoria, Australia; School of Public Health and Preventive Medicine, Monash University, Melbourne, Victoria, Australia; Department of Infectious Diseases, Alfred Health and Monash University, Melbourne, Victoria, Australia; Melbourne School of Population and Global Health, University of Melbourne, Melbourne, Victoria, Australia; Disease Elimination Program, Burnet Institute, Melbourne, Victoria, Australia; School of Public Health and Preventive Medicine, Monash University, Melbourne, Victoria, Australia; Department of Infectious Diseases, Alfred Health and Monash University, Melbourne, Victoria, Australia

**Keywords:** cost-effectiveness, hepatitis C, point-of-care

## Abstract

**Background:**

Eliminating hepatitis C virus requires efficient testing and treatment strategies. We evaluated cost-effectiveness of alternative hepatitis C virus diagnosis and treatment initiation approaches for treatment-naive people who inject drugs attending Australian community settings.

**Methods:**

We compared 7 strategies differing by use of antibody screening, laboratory, and/or point-of-care tests, and point of treatment commencement. Outcomes were treatment initiation and completion. We considered costs from a healthcare sector perspective at a 1-year time horizon. We used decision analytical models parameterized with publicly available estimates.

**Results:**

Standard of care laboratory antibody then RNA testing on separate samples was cheapest but least effective. Laboratory antibody then reflex RNA testing on 1 sample provided higher effectiveness and was the only strategy to reduce average cost per completion ($6141 2023AUD; 95% confidence interval, $3924–$10,382). Combined point-of-care antibody and RNA testing, point-of-care RNA alone, and point-of-care antibody with immediate treatment initiation in turn provided incremental improvements in completion at higher average costs per completion (point estimates: $6976–$11 707AUD). Changes in treatment uptake of at least 16 points were required to achieve equivalence between reflex laboratory and point-of-care strategies. Although treatment of nonviremic individuals contributed to higher costs of point-of-care strategies, reflex laboratory testing remained less costly per completion at generic medication costs.

**Conclusions:**

Reflex RNA testing was the most efficient strategy and can be implemented within the existing Australian laboratory framework. Point-of-care approaches may provide additional benefit at higher near-term costs. Studies accounting for transmission and disease sequelae are needed to understand cost-effectiveness in the longer term.

## Teaser

Evaluated cost-effectiveness of alternative approaches to HCV diagnosis and treatment initiation for treatment-naive people who inject drugs in Australian community settings. Reflex RNA testing resulted in lowest cost per completion. Point-of-care approaches provided additional benefit at higher near-term costs.

Diagnosis is a prerequisite to accessing treatment for hepatitis C, yet most people living with chronic hepatitis C virus (HCV) infection are unaware that they are infected [1]. While progress toward elimination has been made in some countries, persistent and even growing incidence in others underscores the need to improve context-specific approaches to testing and linkage to care [2].

In Australia, improvements in rates of diagnosis and treatment followed the introduction of subsidized direct-acting antivirals (DAAs) in 2016, with flow-on reductions in disease burden [3]. By the end of 2022, an estimated 81% of individuals living with chronic hepatitis C in Australia were diagnosed antibody-positive, and viremia declined from 51% to 12% in people who inject drugs surveyed between 2015 and 2022 [4]. Management of hepatitis C infection has effectively shifted from specialist care to primary care and drug and alcohol services [5]. Nevertheless, recent deceleration in progress towards elimination targets has prompted calls for innovative strategies to engage the remaining undiagnosed population [6, 7].

Optimizing the HCV care cascade for people who inject drugs is central to hepatitis C elimination [8]. Various interventions have been explored to enhance linkage to care, including education, financial incentives, peer support, and alternative models of care [9]. This paper focuses on understanding the most efficient approaches to testing and treatment initiation in the context of an increasing variety of diagnostic technologies available in community care settings.

Diagnosis of active HCV infection relies on detection of viral RNA or antigen. Antibodies provide evidence of prior infection and are a useful target for screening in people who have not been treated, given a minority of acute infections clear spontaneously. In most high-income countries, standard of care consists of sequential laboratory HCV antibody and RNA testing of venipuncture samples [10], necessitating multiple healthcare encounters associated with substantial loss to follow-up [10]. Although guidelines recommend reflex HCV RNA testing whenever an individual has a detectable antibody test (“reflex testing”), this practice is not yet routine, meaning many patients must return for a second venipuncture before they can obtain a diagnosis of active HCV infection [3].

In contrast to laboratory assays, point-of-care testing can provide rapid results within the same visit without the need for venipuncture, thereby reducing barriers to care faced by people who inject drugs [9]. Although observational evidence suggests that point-of-care approaches improve rates of treatment initiation [11], they are also more expensive at present [12] and marginally less accurate than laboratory tests [13, 14]. Both point-of-care HCV RNA and antibody tests are available, allowing for implementation of strategies that combine point-of-care screening with either point-of-care or laboratory RNA confirmation before starting treatment.

Availability of point-of-care testing has raised the possibility of achieving testing and treatment initiation within the same visit [15], yet at present point-of-care RNA tests require around an hour to return a result, potentially limiting acceptability of this model of care [16]. To further increase treatment uptake, another same-day clinical approach uses HCV point-of-care antibody testing, with immediate treatment initiation for eligible antibody-positive individuals and follow up within 1 week to stop therapy in those who are RNA negative on confirmatory laboratory testing [17, 18]. This approach to assessment and treatment (referred to as “immediate treatment” throughout this paper) is analogous to presumptive treatment of high-risk asymptomatic contacts of individuals with certain sexually transmitted infections [19] and is currently being assessed in a randomized controlled trial [17].

In this context, we conducted a modeled cost-effectiveness evaluation comparing alternative pathways to hepatitis C diagnosis and treatment initiation. We focused on treatment-naive people who inject drugs attending community primary care settings as an important real-world context where point-of-care assays will be deployed. By synthesizing existing evidence and incorporating economic considerations, our study aims to provide insights to guide immediate policy decisions, as well as a framework applicable to alternative contexts.

## METHODS

### Evaluation Scope

#### Target Population and Setting

We designed the evaluation for a target population of HCV treatment-naive individuals who inject drugs and access care in Australian community primary care settings. These settings include general practices, community health centers, drug treatment services, and needle and syringe provision sites. About 0.6% of 15- to 64-year-olds in Australia inject drugs [20]. Among those participating in the 2022 national sentinel Illicit Drug Reporting System, the mean age was 46 years, two-thirds identified as male, 87% were unemployed, and 16% had no fixed address; methamphetamine followed by heroin were the drugs most likely to be injected on a regular basis [21]. Among respondents to the 2022 Australian Needle and Syringe Program Survey, 32% had evidence of anti-HCV antibody, 12% had detectable HCV RNA, and 13% reported previous treatment for hepatitis C [22].

#### Interventions

**Figure 1.**
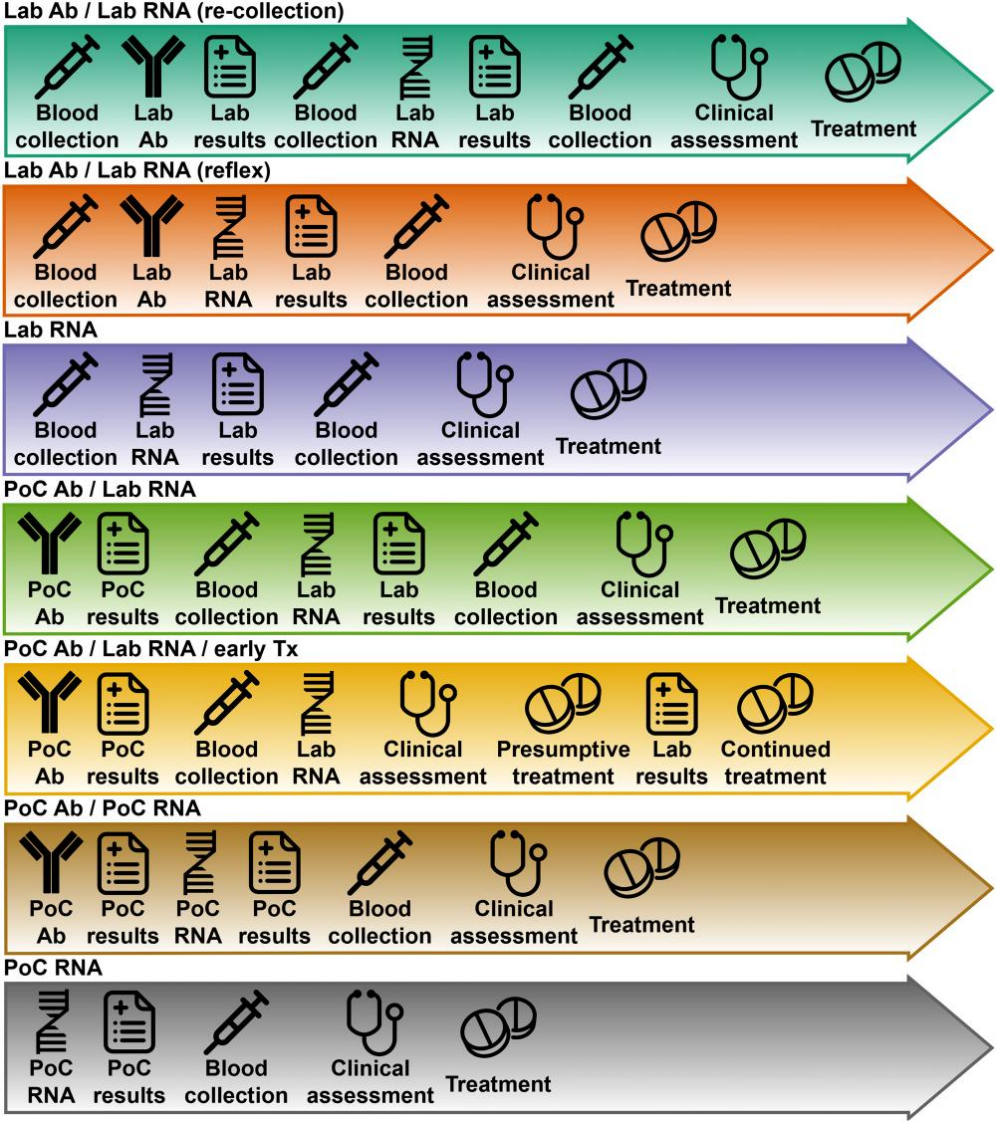
HCV diagnosis and treatment initiation strategies included in evaluation. See Supplementary figure 1 for full model structure. S1.a – Lab Ab / Lab RNA (re-collection): antibody followed by RNA testing (if antibody positive) in laboratory on venipuncture samples requiring collection of second venipuncture sample for RNA testing. Clinical assessment and treatment initiation based on RNA positivity. S1.b – Lab Ab / Lab RNA (reflex): antibody followed by RNA testing (if antibody positive) in laboratory on single venipuncture sample. Clinical assessment and treatment initiation based on RNA positivity. S1.c – Lab RNA: RNA testing in laboratory on venipuncture sample. Clinical assessment and treatment initiation based on RNA positivity. S2.a – PoC Ab / Lab RNA: point-of-care antibody testing on fingerstick sample followed by venipuncture for RNA testing in laboratory (if antibody positive). Clinical assessment and treatment initiation based on RNA positivity. S2.b – PoC Ab / Lab RNA / early Tx: point-of-care antibody testing on fingerstick sample followed by venipuncture for RNA testing in laboratory (if antibody positive). Clinical assessment and presumptive treatment initiation for those who are antibody positive, with review of laboratory results at 1 week to continue (RNA positive) or cease treatment (RNA negative). S3.a – PoC Ab / PoC RNA: point-of-care antibody testing on fingerstick sample followed by point-of-care RNA testing on fingerstick sample (if antibody positive), and laboratory RNA testing if point-of-care RNA result is invalid. Clinical assessment and treatment initiation based on RNA positivity. S3.b – PoC RNA: point-of-care RNA testing on fingerstick sample, and laboratory RNA testing if point-of-care RNA result is invalid. Clinical assessment and treatment initiation based on RNA positivity. Abbreviations: Ab: antibody; HCV: hepatitis C virus; Lab: laboratory; PoC: point-of-care; Tx: treatment.

We evaluated seven strategies describing broad approaches to care following consent to HCV screening through diagnosis and treatment initiation (Figure 1). Strategies reflected either current standard of care [10] or alternative strategies under evaluation in Australian settings [17, 23]. The strategies varied based on whether they involved an antibody screening test before RNA testing confirmation; used laboratory or point-of-care testing; and initiated treatment based on confirmed viremia, or a positive antibody test (with subsequent RNA results determining treatment continuation).

#### Outcomes

We estimated the number of viremic individuals starting and completing treatment per 1000 screened, and the costs. We evaluated cost-effectiveness using the net monetary benefit approach [24] and willingness to pay thresholds at $100 increments up to $250 000.

#### Health Economic Parameters

We adopted a healthcare sector perspective [25] and a time horizon of 1 year without applying a discount rate.

#### Ethics and Reporting

We reported the evaluation in accordance with the Consolidated Health Economic Evaluation Reporting Standards 2022 statement [26] (Supplementary material). All data used are in the public domain and human research ethics committee review was not required.

### Model

We used decision tree analytical models to estimate costs and effects for each strategy (Supplementary figure 1). In designing the models, we were guided by good practice recommendations provided by Philips et al. [27]. We assumed uniform screening uptake across strategies. For each strategy, HCV RNA and antibody status were determined based on population epidemiological parameters. Outcome probabilities and associated costs were then determined using a care cascade model for each strategy. Loss to follow-up was considered at every testing stage and before or after treatment initiation. We implemented analyses in R version 4.1.2 [28] and have made code available via Github (https://github.com/ChrisBailie/HCV-CE).

We assumed point-of-care testing used fingerstick samples with the OraQuick point-of-care HCV antibody test (OraSure; Pennsylvania, USA) [29] and Xpert HCV VL Fingerstick assay (Cepheid; California, USA) [30]. The latter produces invalid results if an insufficient volume of blood is added to the cartridge. We assumed that individuals with invalid point-of-care RNA results reverted to standard laboratory RNA testing.

#### Parameters

##### Transition Probabilities

Parameter estimates are provided in Table 1. We selected epidemiological, loss to follow-up, and test performance parameters based on estimates from surveys [22, 31], observational clinical studies [32], analysis of surveillance data [33], and test validation studies [13, 14] identified through a literature review. Empirically derived estimates of the care cascade for strategies combining point-of-care antibody testing and laboratory RNA testing were not available; we therefore selected estimates based on expected difference in loss to follow-up compared with standard of care underlying sample size calculations for a randomized controlled trial [17].

**Table 1.** Model Parameters

Input Parameter	Estimate	Distribution For Sensitivity Analysis	Sources	Figure Reference (Supplementary Figure 1)
**Transition probabilities**				
Probability RNA present	200/1653 = 12%	β (α = 201, β = 1454)^a^	[22]	p_1_
Probability antibody present given absent RNA	354/1524 = 23%	β (α = 355, β = 1171)^a^	[22]	p_2_
Sensitivity of OraQuick point-of-care HCV antibody fingerstick	99.5% (95% CI, 98.9%–99.8%)	β (α = 1000, β = 6)^b^	[13]	p_3_
Specificity of OraQuick point-of-care HCV antibody fingerstick	99.8% (95% CI, 99.6–99.9)	Beta (α = 1000, β = 3)^b^	[13]	p_4_
Sensitivity of laboratory Ab test	100%	-	Assumption	p_5_
Specificity of laboratory Ab test	100%	-	Assumption	p_6_
Sensitivity of Cepheid Xpert HCV VL fingerstick	99% (95% CI, 97%–99%)	β (α = 287.4, β = 3.9)^b^	[14]	p_7_
Specificity of Cepheid Xpert HCV VL fingerstick	99% (95% CI, 96%–100%)	β (α = 160.2, β = 2.6)^b^	[14]	p_8_
Probability of invalid result with Cepheid Xpert HCV VL fingerstick	6% (95% CI, 3%–11%)	β (α = 9.9, β = 139.8)^b^	[14]	p_9_
Sensitivity of laboratory RNA test	100%	-	Assumption	p_10_
Specificity of laboratory RNA test	100%	-	Assumption	p_11_
Probability laboratory RNA performed given laboratory Ab + ve (*S1.a*)	13 937/19 914 = 70%	β (α = 13,938, β = 5978)^a^	[33]	p_12_
Probability laboratory RNA performed given PoC Ab + ve (*S2.a*)	90% (95% CI, 80%–95%)	β (α = 57.1, β = 7.2)^b^	Assumption [17]	p_13_
Probability laboratory RNA performed and starts treatment given PoC Ab + ve (*S2.b*)	90% (95% CI, 80%–95%)	β(α = 57.1, β = 7.2)^b^	Assumption [17]	p_14_
Probability receives RNA result and continues treatment given immediate treatment commenced and laboratory RNA positive (*S2.b*)	75% (95% CI: 50%–90%)	β (α = 12.8, β = 4.9)^b^	Assumption [17, 18]	p_15_
Probability PoC RNA performed given PoC Ab + ve (*S3.a*)	140/150 = 93%	β (α = 141, β = 11)^a^	[32]	p_16_
Probability starts treatment given PoC RNA + ve (*S3.a, S3.b*)	44/70 = 63%	β (α = 45, β = 27)^a^	[32]	p_17_
Probability laboratory RNA performed given PoC RNA invalid (*S3.a, S3.b*)	90% (95% CI, 80%–95%)	β (α = 57.1, β = 7.2)^b^	Assumption	p_18_
Probability starts treatment given laboratory RNA + ve (*S1.a, S1.b, S1.c, S2.a, S3.a, S3.b*)	6876/14 630 = 47%	β (α = 6877, β = 7755)^a^	[31]	p_19_
Probability completes treatment given treatment started (all strategies)	26/44 = 59%	β (α = 27, β = 19)^a^	[32]	p_20_
**Costs**				
OraQuick point-of-care HCV antibody fingerstick	$43.09 (2023 AUD)	Uniform (± 20%)	[36]	c_1_
Cepheid Xpert HCV VL fingerstick	$145.2 (2023 AUD)	Uniform (± 20%)	[12]	c_2_
Laboratory HCV antibody	$15.65 (2023 AUD)	-	MBS 69 475 [37]	c_3_
Laboratory HCV RNA	$92.2 (2023 AUD)	-	MBS 69 499 [37]	c_4_
Clinical assessment (excluding venipuncture)	$173.35 (2023 AUD)	-	…	c_5_
Hepatitis A and B virus serology	$29.25 (2023 AUD)	-	MBS 69 478 [37]	…
HIV serology	$15.65 (2023 AUD)	-	MBS 69384 [37]	…
Full blood examination	$16.95 (2023 AUD)	-	MBS 65070 [37]	…
Liver function tests + eGFR	$17.7 (2023 AUD)	-	MBS 66512 [37]	…
International Normalized Ratio	$13.7 (2023 AUD)	-	MBS 65120 [37]	…
Clinician time	$80.10 (2023 AUD)	-	MBS 36 [37]	…
Venipuncture for laboratory tests	$9.95 (2023 AUD)	Uniform (+ 0%–50%)	MBS 73938 MBS 74998 [37]	c_6_
One-wk treatment only (*S2.b*)	$1125 (2023 AUD)	-	[53]	c_7_
Partial treatment course	$5998 (2023 AUD)	Uniform (+/− 20%)	[53]	c_8_
Completed treatment course	$13,495 (2023 AUD)	-	[53]	c_9_

^a^β parameters derived from reported counts of trials and successes.

^b^β parameters selected to optimize mode to reported or assumed point estimate and 2.5%/97.5% quantiles to 95% confidence/certainty interval.

Abbreviations: Ab: antibody; CI: confidence/credible interval; HCV: hepatitis C virus; PoC: point-of-care.

##### Costs

We estimated all costs in 2023 Australian dollars. We converted costs expressed in international currencies into Australian dollars for the equivalent year according to the Purchasing Power Parities [34] and adjusted for inflation using the Australian Bureau of Statistics Consumer Price Index [35].

We included economic costs associated with diagnosis, assessment, and treatment after scale-up. We used applicable published estimates, where available, and otherwise took an ingredients-based costing approach. For diagnosis, we used published estimates for point-of-care tests [12, 36], and Medicare Benefits Schedule (MBS) rebates as proxies for the cost of laboratory tests [37]. We estimated costs of clinical assessment from the sum of MBS rebates for attendance and laboratory testing components recommended according to clinical guidelines, assuming fibrosis assessment was based on AST to Platelet Ratio Index [10, 37].

For the treatment completion outcome, we did not include costs of complete treatment in viremic individuals because these were assumed to be the same in all strategies and were considered in original recommendations for subsidizing DAAs [38]. We did, however, include “wasted” costs of partial treatment and of treating nonviremic individuals. This approach was necessary to account for varying treatment costs due to presumptive treatment based on HCV antibody results as well as imperfect test performance.

Prices paid by the Australian government for drug therapies are generally published via Pharmaceutical Benefits Scheme (PBS) schedule; however, DAAs for hepatitis C are subject to confidential pricing agreements, implying that the actual price is less than what is published [38]. The Australian government committed $1.2 billion for unlimited DAA treatment courses between 2016 and 2021 [39], and approximately 88 920 people were treated over this period [4], yielding an estimated average cost of $13 495 per course. We assumed that subsequent negotiations resulted in a similar price per course and adopted this value (without adjusting for inflation) for the cost of treatment under our base-case analysis. This represents a 63% discount to the current Australian list price. We performed sensitivity analyses examining alternative assumptions.

We assumed that medication costs for partial treatment were proportionate to the quantity dispensed and cost of a full course. Based on 2022 treatment uptake data [5], we assumed that two-thirds of people who started but did not complete treatment would do so after being dispensed 4 weeks’ worth of medication and another third after 8 weeks; estimated costs of partial treatment as the weighted average of costs under these scenarios. We did not cost follow-up appointments during treatment because these are not routinely required under current guidelines for individuals without comorbidities [10].

#### Sensitivity Analyses

##### Probabilistic

We estimated cost-effectiveness in the context of uncertainty following the expected net loss approach [24] using 1000 sets of sampled parameters. We modeled transition probability uncertainty with β distributions and cost uncertainty with uniform distributions. Where we derived transition probability point estimates from a binomial experiment in a single study, we parameterized β distributions using the formulas *α* = *k* + 1 and *β* = *n* − *k* + 1, where *k* is the number of successes and *n* is the number of trials. Where we derived transition probability point estimates from meta-analyses, we obtained β distribution parameters from reported point estimates and confidence intervals using an optimization algorithm [40]. Where little information was available on parameter uncertainty, we selected parameters to provide conservative estimates of uncertainty.

##### Deterministic

We performed 4 deterministic sensitivity analyses addressing alternative scenarios. First, we repeated the base case analysis varying RNA prevalence from 2% to 50% using a range of plausible corresponding values for antibody prevalence derived from Australian Needle and Syringe Program Surveys conducted between 2015 and 2022 (Supplementary Figure 2) [41]. Second, we simultaneously varied loss to follow-up parameters specific to the immediate treatment strategy, which were based on expert opinion rather than empiric estimates, to produce conservative upper and lower bounds on cost-effectiveness of this strategy. Third, for each strategy, we independently adjusted the set of parameters determining loss to follow-up before RNA-confirmed treatment initiation—or continuation at 1 week for the immediate treatment strategy—by multiplying all parameters in the set by the same scale constant, up to a ceiling of 1. We performed a similar procedure for loss to follow-up between RNA-confirmed treatment initiation and treatment completion. Finally, we repeated the base analysis with adjusted treatment costs to reflect the sofosbuvir/velpatasvir price listed on the PBS ($36 111 per 12-week course), as well as an estimated generic price [42].

## RESULTS

**Figure 2.**
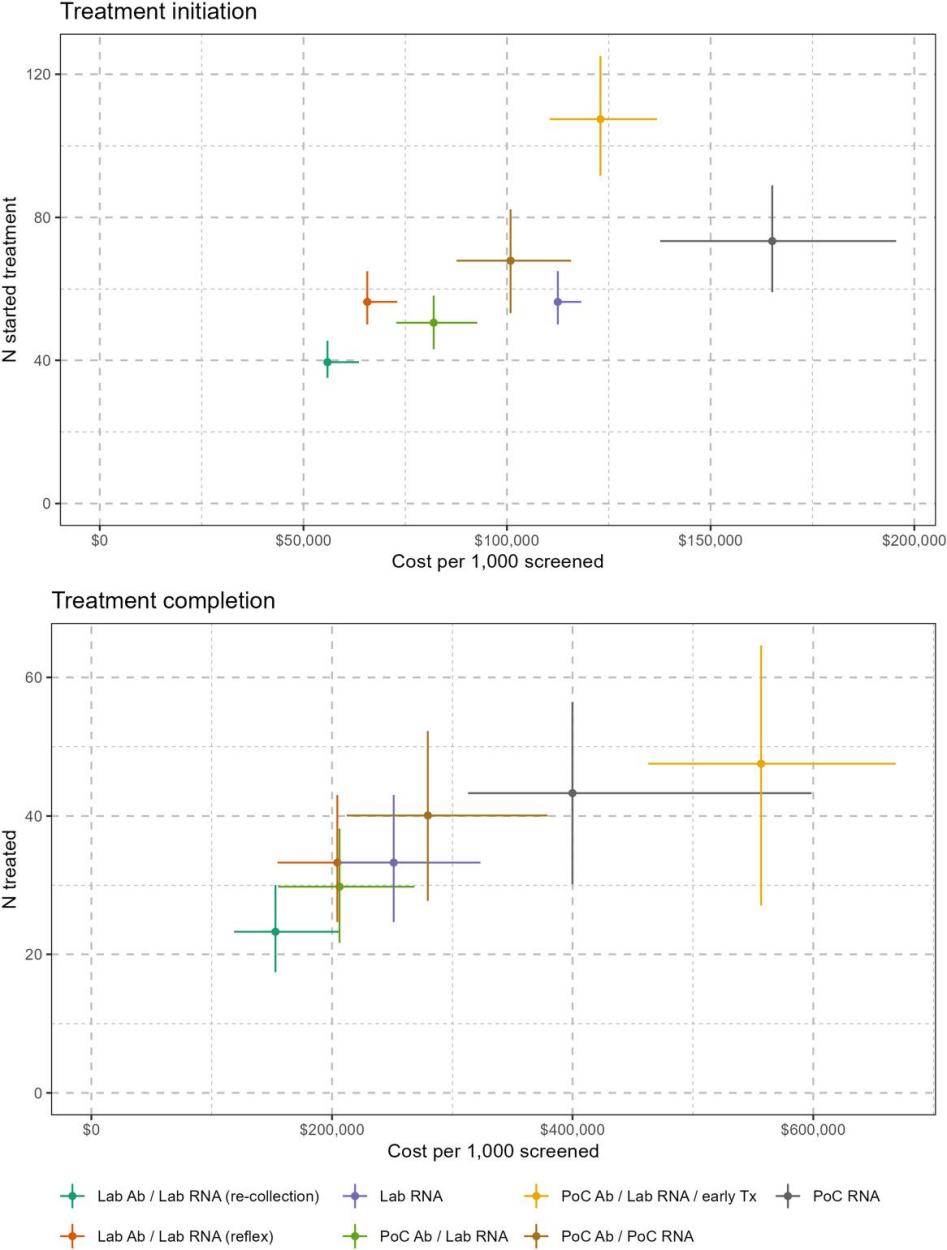
Estimated cost-effectiveness of hepatitis C treatment initiation strategies for treatment-naive people who inject drugs (costs in 2023 AUD). Point estimates obtained under base-case assumptions and 95% uncertainty intervals from 1000 probabilistic draws. Abbreviations: Ab: antibody; Lab: laboratory; PoC: point-of-care; Tx: treatment.

Standard of care (Lab Ab / Lab RNA [re-collection]) was the cheapest and least effective strategy, resulting in 23 (95% CI, 17–30) treatment completions per 1000 screened at a total cost of $153 000 (95% CI, $118 600–$206 600), or average cost per completion of $6568 (95% CI, $4323–$11 016), under the base-case assumptions (Figure 2, Table 2). Addition of reflex RNA testing (Lab Ab / Lab RNA [reflex]) resulted in an additional 10 (95% CI, 7–13) treatment completions per 1000 screened and the lowest average costs per treatment completion ($6141; 95% CI, $3924–$10 382).

**Table 2.** Estimated Cost-effectiveness of Hepatitis C Treatment Initiation Strategies for Treatment-naive People Who Inject Drugs (Costs in 2023 AUD)

	Average			Incremental to Standard of Care			Willingness-to-pay Per Additional Effect For Which Strategy:	
Strategy	Cost per 1000 Screened	Effects per 1000 Screened (Completions or Initiations)	Cost-effectiveness Ratio	Cost per 1000 Screened	Effects per 1000 Screened (Completions or Initiations)	Cost-Effectiveness Ratio	Maximizes Net Monetary Benefit	Minimizes Expected Net Loss
**Treatment initiation**								
Lab Ab / Lab RNA (re-collection)	$55 900 ($55 300–$63 600)	39 (35–45)	$1416 ($1321–$1682)	•	•	•	$0–$500	$0–$500
Lab Ab / Lab RNA (reflex)	$65 700 ($64 600–$73 000)	56 (50–65)	$1164 ($1084–$1348)	$9800 ($8500–$10 400)	17 (15–20)	$578 ($496–$603)	$600—$1100	$600–$1000
PoC Ab / Lab RNA / early Tx	$123 000 ($110 500–$136 800)	107 (92–125)	$1144 ($1000–$1325)	$67 100 ($51 200–$76 600)	68 (55–80)	$987 ($784–$1163)	≥ $1200	≥ $1100
PoC Ab / PoC RNA	$100 900 ($87 600–$115 700)	68 (53–82)	$1486 ($1213–$1906)	$45 000 ($28 200–$56 600)	28 (16–40)	$1583 ($930–$2674)	•	•
PoC Ab / Lab RNA	$82 000 ($72 800–$92 700)	51 (43–58)	$1623 ($1410–$1911)	$26 100 ($13 200–$33 300)	11 (6–14)	$2365 ($1354–$3760)	•	•
Lab RNA	$112 500 ($112 300–$118 200)	56 (50–65)	$1994 ($1785–$2287)	$56 600 ($54 200–$57 400)	17 (15–20)	$3345 ($2832–$3756)	•	•
PoC RNA	$165 100 ($137 600–$195 500)	73 (59–89)	$2250 ($1743–$3008)	$109 200 ($79 200–$136 000)	34 (21–47)	$3220 ($2047–$5537)	•	•
**Treatment completion**								
Lab Ab / Lab RNA (re-collection)	$153 000 ($118 600–$206 600)	23 (17–30)	$6568 ($4323–$11 016)	•	•	•	$0–$5100	$0–$5100
Lab Ab / Lab RNA (reflex)	$204 400 ($154 700–$277 900)	33 (25–43)	$6141 ($3924–$10 382)	$51 400 ($35 500–$71 400)	10 (7–13)	$5147 ($3015–$8904)	$5200–$11 000	$5200–$12 400
PoC Ab / PoC RNA	$279 600 ($212 200–$379 300)	40 (28–52)	$6976 ($4667–$11 708)	$126 600 ($82 200–$189 000)	17 (9–25)	$7544 ($4968–$13 646)	$11 100–$37 000	$12 500–$43 500
PoC Ab / Lab RNA / early Tx	$556 700 ($462 900–$668 500)	48 (27–65)	$11 707 ($8154–$19 880)	$403 700 ($326 200–$468 800)	24 (7–37)	$16 641 ($10 674–$50 329)	≥ $37 100	≥ $46 900
PoC Ab / Lab RNA	$206 200 ($155 200–$268 400)	30 (22–38)	$6919 ($4480–$11 211)	$53 200 ($29 300–$70 400)	7 (3–9)	$8176 ($4885–$13 870)	•	•
Lab RNA	$251 200 ($201 600–$323 500)	33 (25–43)	$7549 ($5081–$12 235)	$98 200 ($81 900–$118 000)	10 (7–13)	$9838 ($6751–$15 141)	•	•
PoC RNA	$399 900 ($313 000–$598 600)	43 (30–56)	$9235 ($6501–$16 311)	$247 000 ($176 200–$427 400)	20 (12–29)	$12 340 ($8280–$24 822)	•	•

Point estimates obtained under base-case assumptions and 95% uncertainty intervals from 1000 probabilistic draws.

Abbreviations: Ab, antibody; Lab, laboratory; PoC, point-of-care; Tx, treatment.

Point-of-care based strategies were more effective than purely laboratory-based strategies but more costly on a per treatment completion basis. The 2-step point-of-care *(*PoC Ab / PoC RNA) strategy resulted in 17 (95% CI, 9–25) additional completions compared to standard of care at an average cost per completion of $6976 (95% CI, $4667–$11 708), and the point-of-care RNA alone (PoC RNA) in 20 (95% CI, 12–29) additional completions at an average cost of $9235 (95% CI, $6501–$16 311). The immediate treatment (PoC Ab / Lab RNA / early Tx) strategy was most effective, resulting in an additional 24 (95% CI, 7–37) treatment completions compared to standard of care at an average cost of $11 707 (95% CI, $8154–$19 880).

The most efficient set of strategies for treatment completion consisted of reflex laboratory testing, the 2-step point-of-care strategy and the immediate treatment strategy at willingness to pay thresholds of $5200, $11 100, and $37 100, respectively (Supplementary figure 3). For the treatment initiation outcome, the immediate treatment strategy dominated other point-of-care approaches and was preferred to reflex laboratory testing at willingness to pay thresholds above $1200 per treatment initiation. Accounting for total parameter uncertainty, willingness to pay thresholds estimated using a probabilistic expected net loss approach were similar to the base-case estimates for treatment initiation, but slightly higher for treatment completion (Table 2 and Supplementary figure 4).

**Figure 3.**
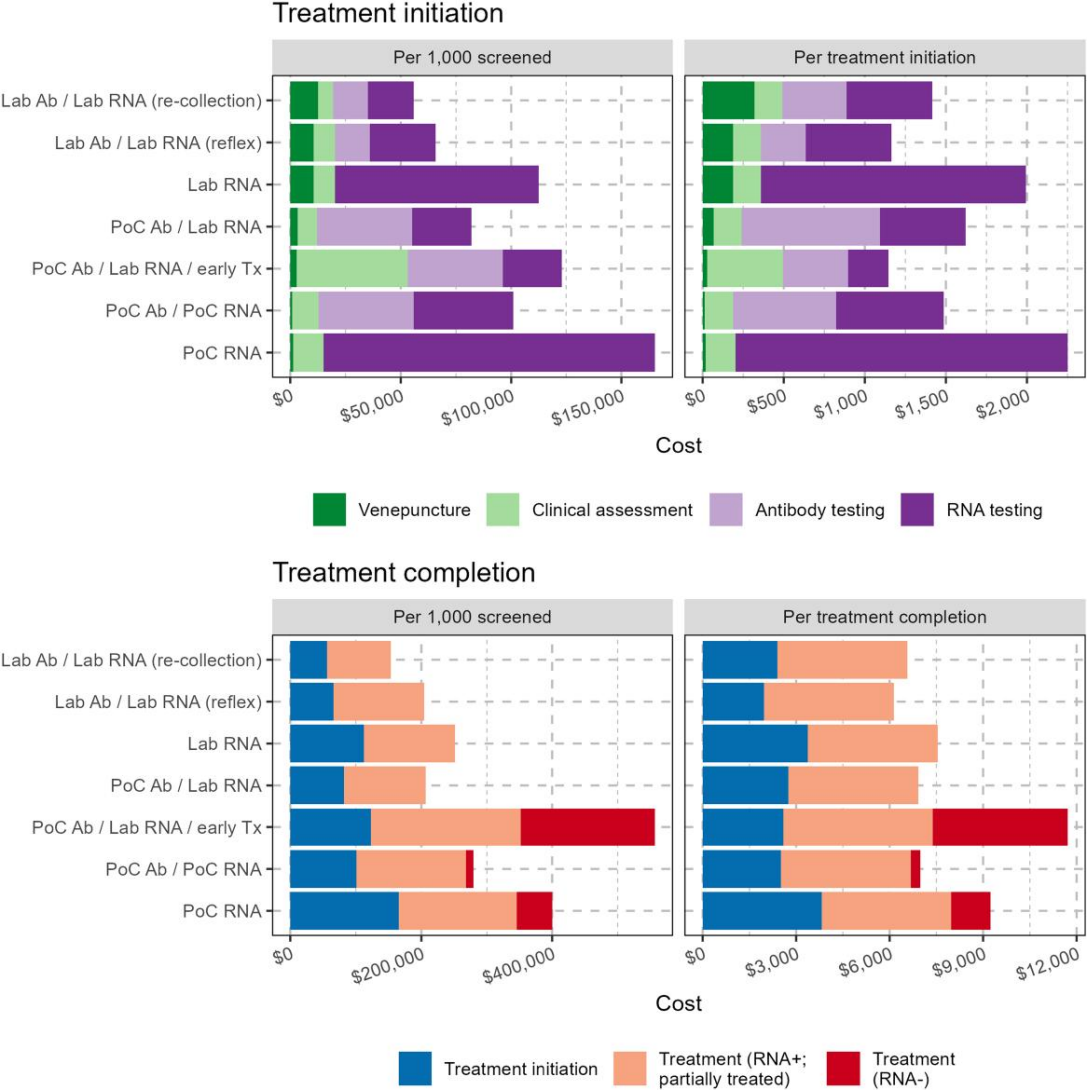
Estimated costs of hepatitis C treatment initiation strategies for treatment-naive people who inject drugs (2023 AUD). Top: costs of treatment initiation. Bottom: overall costs of treatment completion (excluding costs of complete treatment for viremic individuals). Abbreviations: Ab: antibody; Lab: laboratory; PoC: point-of-care; Tx: treatment.

Overall costs were driven heavily by costs of treatment (Figure 3). Differences in cost per treatment completion for strategies where treatment was commenced after laboratory RNA confirmation were due to differences in costs of diagnosis and assessment, to which RNA testing was generally the greatest contributor. For strategies relying on point-of-care RNA confirmation, greater costs per treatment completion were primarily due to costs of treating nonviremic individuals with false-positive RNA results. The higher relative costs of the immediate treatment strategy for treatment completion were partly attributable to medication costs of presumptive treatment of viremic individuals subsequently lost to follow up, but more so to presumptive treatment of nonviremic individuals while awaiting RNA results—for every viremic individual started on treatment another 1.7 nonviremic individuals were treated presumptively.

As HCV prevalence increased, margins in average cost-effectiveness between point-of-care testing and less costly laboratory-based strategies reduced (Supplementary figure 5), as did the willingness to pay thresholds at which point-of-care and immediate treatment strategies were preferred (Supplementary figure 6). Conversely, at low HCV prevalences, particularly with lower antibody prevalence, strategies relying solely on RNA testing were relatively less cost-effective (Supplementary figures 7–8).

Setting loss to follow-up parameters for the immediate strategy at upper and lower certainty bounds resulted in optimistic and pessimistic estimates of average cost per treatment completion of $10 093 and $16 599, respectively (Supplementary figure 9). The strategy remained part of the treatment completion net monetary benefit frontier, providing loss to follow-up between commencing immediate treatment and continuing treatment at 1 week was ≤20% (Supplementary figure 10).

Changes in treatment uptake of at least 16 percentage points were required to achieve equivalent cost per completion between reflex RNA testing and point-of-care strategies (Supplementary figure 11). Due largely to costs of partial treatment, only small differences in treatment completion (given treatment initiation) between strategies were required to achieve equivalent cost per completion between standard of care and reflex RNA testing (2 percentage points) and between reflex RNA testing and the 2-step point-of-care strategy (4 percentage points; Supplementary figure 12).

Setting treatment costs to PBS indicated prices increased willingness to pay threshold at which point-of-care strategies were preferred to laboratory-based strategies from $11 100 to $21 000 per completion (Supplementary table 1). At estimated generic prices, the immediate treatment strategy dominated other point-of-care strategies.

## DISCUSSION

Our findings suggest that reflex laboratory testing, point-of-care antibody then RNA testing, and point-of-care antibody testing with immediate treatment and laboratory RNA confirmation may all be cost-effective alternative approaches to hepatitis C testing and treatment initiation for people who inject drugs attending Australian community care settings, depending on the value placed on treating an additional person. Reductions in prevalence favor strategies incorporating an antibody testing step as a mechanism to reduce costly RNA testing. At higher medication costs, misdiagnosis or presumptive treatment of nonviremic individuals and loss to follow-up after treatment initiation are important determinants of cost-effectiveness.

Reflex laboratory testing was the only strategy that reduced average cost per treatment completion compared with standard of care. Single-sample reflex testing has been implemented elsewhere with negligible change in accuracy and very few samples of insufficient volume (0.4%) [43]. Use of reflex testing was identified as a key area for action in Australia's Sixth National Hepatitis C Strategy 2023–2030 [44] and has been recommended in clinical guidelines and endorsed as a pathologist determinable test (ie, not requiring a specific clinician request for samples that are antibody positive or indeterminate) since 2022 [10, 45]. The effect of these initiatives on historically poor rates of follow-up RNA testing [3, 33] are yet to be adequately described, but anecdotes suggest uptake has been limited. In addition to monitoring uptake, more needs to be done to encourage implementation of reflex laboratory testing, for example by modifying testing rebates to provide incentives for pathology providers to participate.

Strategies including an antibody testing step are relatively more efficient as RNA prevalence decreases, so are particularly relevant among populations or locations nearing elimination. Point-of-care antibody tests for HCV are acceptable [46], relatively simple to perform and provide results in a short timeframe [29], allowing for higher throughput than point-of-care RNA testing alone [47]. Point-of-care antibody testing may be coupled with either point-of-care or laboratory RNA confirmation, although our results suggest the latter is efficient only if treatment is started immediately, prior to the RNA result becoming available.

Coupling point-of-care antibody with point-of-care RNA testing can facilitate rapid diagnosis and treatment initiation on the same visit [15], but there are implementation issues associated with the complexity of the point-of-care RNA testing. Point-of-care tests can achieve diagnosis without the need for venipuncture, improving acceptability for people who inject drugs. However, while treatment strategies that avoid venipuncture are possible [48], venipuncture for biochemistry, hematology, and serology is still recommended for pretreatment assessment under current guidelines [10]. Point-of-care RNA testing is also associated with up-front costs of the testing platform, staff training, and additional space requirements. The wait time of around 1 hour for a result may still be too long for some people who inject drugs, resulting in loss to the care cascade, undermining some benefits of rapid results [16]. Invalid results are not uncommon in validation studies [14], requiring retesting or referral to another pathway. Furthermore, as hepatitis C surveillance in Australia generally relies on laboratory reporting, testing conducted outside laboratories requires implementation of systems to ensure that positive results are notified in a complete and timely manner.

On the other hand, barring high drug costs, point-of-care antibody testing coupled with existing laboratory RNA testing and a novel approach to treatment initiation may maintain the effectiveness benefits of same-day treatment initiation without the complexities associated with point-of-care RNA testing. Presumptive treatment initiation based on epidemiologic risk is a common strategy for managing sexually transmitted infections [19]; however, such conditions typically require shorter treatment courses at lower cost. The higher costs of the immediate treatment strategy in our evaluation were due to medication costs of presumptive treatment. If real-world effectiveness approaches that assumed for our evaluation, and medication costs are substantially lower than our assumptions, or if a suitable test with higher specificity for viremia becomes available (eg, a HCV antigen test, super-fast RNA test), this strategy is likely to be efficient for a wide range of HCV prevalence. Mechanisms to fund presumptive treatment would be needed in Australia as current PBS prescribing criteria require evidence of HCV RNA.

Several previous modeling studies have estimated cost-effectiveness of alternative testing strategies in heterogenous international settings, generally favoring point-of-care approaches [49, 50]. One previous evaluation has been conducted for the Australian context [12]; Shih et al. used decision trees to estimate cost effectiveness of point-of-care approaches in several settings (prisons, needle and syringe programs, drug treatment clinics), finding on average lower costs per treatment initiation for point-of-care approaches compared to standard of care [12]. Although we adopted the estimate of point-of-care RNA testing cost used in that study, other differences such as in HCV prevalence, point-of-care RNA specificity and treatment uptake, and the approach to managing invalid point-of-care RNA results, were sufficient to make standard of care on average marginally cheaper per treatment initiation in our evaluation [12]. Our study suggests that preference for either laboratory or point-of-care approaches on a cost-per-treatment-completion basis is sensitive to relatively small changes in assumed loss to follow-up. This study builds on previous work by evaluating a wider range of strategies, including reflex laboratory RNA testing, and by accounting for costs of treatment.

Because the cost-effectiveness benefits of point-of-care approaches are driven by higher treatment uptake, it is important to consider how improvements observed in research studies might translate into routine practice. Existing studies of the effect of point-of-care testing on treatment uptake are generally observational or quasi-experimental and of low quality [11]. It is often impossible to distinguish benefits of the testing approach from other bundled interventions; for example, provision of peer educators or dedicated viral hepatitis nurses [15, 47]. Furthermore, benefits attributable to point-of-care testing may be negligible in services that have already achieved relatively high levels of treatment uptake through other means [51]. Evidence on the effectiveness of a point-of-care antibody presumptive treatment approach on linkage to care for hepatitis C does not yet exist, and we look forward to results of the QuickStart randomized controlled trial comparing this and other point-of-care strategies to standard of care [17, 18].

Another important question to be addressed by the QuickStart trial is whether the additional people who commence treatment through point-of-care approaches are as likely to complete treatment and achieve sustained virological response. Because of a lack of empiric evidence, an assumption of our base-case analysis was that once infection was confirmed, individuals who started treatment under different strategies were equally likely to finish. However, sensitivity analysis showed that even slightly relaxing this assumption could alter the cost-effective strategy. Strategies with varying loss to follow up in the diagnosis phase could select treatment populations either more or less likely to complete treatment.

Our study has several other important limitations. The short time horizon means that although we consider costs of treatment, we do not consider reduction in disease burden and healthcare costs in individuals treated through the study interventions, or in the wider population because of averted transmission. Accounting for these benefits requires modeling progression of the hepatitis C epidemic, in addition to disease, and is the subject of ongoing work. Doing so would result in strategies capable of achieving higher treatment completion appearing more favorable in the longer term.

Effective prices paid by the Australian government for DAAs remain confidential. If our base-case estimate was below the effective price, our analysis underestimated “wasted” treatment costs associated with point-of-care strategies, and vice versa. Greater transparency would allow more robust health economic evaluation of interventions aiming to improve hepatitis C treatment uptake or adherence.

Our evaluation was targeted towards strategies for treatment-naïve individuals, yet used epidemiologic parameters derived from a sample that also included treatment-experienced people who inject drugs (13%) [41]. Presence of HCV antibody should provide higher specificity for viraemia in the treatment-naïve, suggesting our base-case analysis may have underestimated the relative cost-effectiveness of strategies including an antibody screening step. More generally, variation within community care settings means a one-size-fits-all approach based on parameters selected for the base-case model will not be appropriate for all services. Changes in the screened population including anticipated reduction in HCV prevalence will likely affect the optimal set of strategies. We have made our model readily available to adjust and update.

Although an increasing proportion of people who inject drugs with chronic hepatitis C now report previous treatment [4], individuals with a documented history of HCV antibody positivity do not require rescreening, and should instead be tested for RNA if indicated [10], considering the more straightforward tradeoffs of a point-of-care versus laboratory RNA approach [12]. We did not evaluate strategies using dried-blood spot or HCV core antigen testing, which may be useful for home testing or high-prevalence settings respectively [52]. Finally, our analysis did not address equity concerns, an important consideration in the Australian context where hepatitis C disproportionately impacts Aboriginal and Torres Strait Islander people [3].

Improving testing and linkage to care for people who inject drugs is central to the success of Australia's hepatitis C elimination efforts. Reflex RNA testing can improve efficiency within the existing laboratory testing framework, reducing investment required per treatment completion, and point-of-care approaches can provide additional benefit at higher cost. This evaluation will inform future implementation of alternative testing and treatment initiation strategies.

## Supplementary Material

ofaf514_Supplementary_Data

## Supplementary Data

Supplementary materials are available at *Open Forum Infectious Diseases* online. Consisting of data provided by the authors to benefit the reader, the posted materials are not copyedited and are the sole responsibility of the authors, so questions or comments should be addressed to the corresponding author.
