# Supplementary material for: Cost-effectiveness of Alternative Approaches to Hepatitis C Diagnosis and Treatment Initiation for Treatment-naive People Who Inject Drugs in Australia: A Model-based Economic Evaluation
Source: Open Forum Infect Dis. 2025 Aug 22;12(9):ofaf514. doi: 10.1093/ofid/ofaf514 (PMC12448928; doi:10.1093/ofid/ofaf514)
Supplement: ofaf514_Supplementary_Data [file ofaf514_supplementary_data.docx]

Cost-effectiveness of alternative approaches to hepatitis C diagnosis and treatment initiation for treatment-naive people who inject drugs in Australia: a model-based economic evaluation

Christopher R Bailie, Nick Scott, Alisa E Pedrana, Margaret E Hellard, Joseph S Doyle

Supplementary material

# CHEERS 2022 Checklist

| **Topic** | **No.** | **Item** | **Location where item is reported** |
| --- | --- | --- | --- |
| **Title** |  |  |  |
|  | 1 | Identify the study as an economic evaluation and specify the interventions being compared. | Title |
| **Abstract** |  |  |  |
|  | 2 | Provide a structured summary that highlights context, key methods, results, and alternative analyses. | Abstract |
| **Introduction** |  |  |  |
| **Background and objectives** | 3 | Give the context for the study, the study question, and its practical relevance for decision making in policy or practice. | Background |
| **Methods** |  |  |  |
| **Health economic analysis plan** | 4 | Indicate whether a health economic analysis plan was developed and where available. | Methods |
| **Study population** | 5 | Describe characteristics of the study population (such as age range, demographics, socioeconomic, or clinical characteristics). | Methods |
| **Setting and location** | 6 | Provide relevant contextual information that may influence findings. | Background and methods |
| **Comparators** | 7 | Describe the interventions or strategies being compared and why chosen. | Methods |
| **Perspective** | 8 | State the perspective(s) adopted by the study and why chosen. | Methods |
| **Time horizon** | 9 | State the time horizon for the study and why appropriate. | Methods |
| **Discount rate** | 10 | Report the discount rate(s) and reason chosen. | Methods |
| **Selection of outcomes** | 11 | Describe what outcomes were used as the measure(s) of benefit(s) and harm(s). | Methods |
| **Measurement of outcomes** | 12 | Describe how outcomes used to capture benefit(s) and harm(s) were measured. | Methods |
| **Valuation of outcomes** | 13 | Describe the population and methods used to measure and value outcomes. | N/A |
| **Measurement and valuation of resources and costs** | 14 | Describe how costs were valued. | Methods |
| **Currency, price date, and conversion** | 15 | Report the dates of the estimated resource quantities and unit costs, plus the currency and year of conversion. | Methods |
| **Rationale and description of model** | 16 | If modelling is used, describe in detail and why used. Report if the model is publicly available and where it can be accessed. | Methods |
| **Analytics and assumptions** | 17 | Describe any methods for analysing or statistically transforming data, any extrapolation methods, and approaches for validating any model used. | Methods |
| **Characterising heterogeneity** | 18 | Describe any methods used for estimating how the results of the study vary for subgroups. | N/A |
| **Characterising distributional effects** | 19 | Describe how impacts are distributed across different individuals or adjustments made to reflect priority populations. | N/A |
| **Characterising uncertainty** | 20 | Describe methods to characterise any sources of uncertainty in the analysis. | Methods |
| **Approach to engagement with patients and others affected by the study** | 21 | Describe any approaches to engage patients or service recipients, the general public, communities, or stakeholders (such as clinicians or payers) in the design of the study. | N/A |
| **Results** |  |  |  |
| **Study parameters** | 22 | Report all analytic inputs (such as values, ranges, references) including uncertainty or distributional assumptions. | Results and Table 1 |
| **Summary of main results** | 23 | Report the mean values for the main categories of costs and outcomes of interest and summarise them in the most appropriate overall measure. | Results, Table 2, figures |
| **Effect of uncertainty** | 24 | Describe how uncertainty about analytic judgments, inputs, or projections affect findings. Report the effect of choice of discount rate and time horizon, if applicable. | Results, supplementary material |
| **Effect of engagement with patients and others affected by the study** | 25 | Report on any difference patient/service recipient, general public, community, or stakeholder involvement made to the approach or findings of the study | N/A |
| **Discussion** |  |  |  |
| **Study findings, limitations, generalisability, and current knowledge** | 26 | Report key findings, limitations, ethical or equity considerations not captured, and how these could affect patients, policy, or practice. | Discussion |
| **Other relevant information** |  |  |  |
| **Source of funding** | 27 | Describe how the study was funded and any role of the funder in the identification, design, conduct, and reporting of the analysis | Funding |
| **Conflicts of interest** | 28 | Report authors conflicts of interest according to journal or International Committee of Medical Journal Editors requirements. | Conflicts of interest |

*From:* Husereau D, Drummond M, Augustovski F, et al. Consolidated Health Economic Evaluation Reporting Standards 2022 (CHEERS 2022) Explanation and Elaboration: A Report of the ISPOR CHEERS II Good Practices Task Force. Value Health 2022;25. <doi:10.1016/j.jval.2021.10.008>

## Supplementary tables

**Supplementary table 1.** Estimated cost-effectiveness of hepatitis C treatment initiation strategies for treatment-naive people who inject drugs under varying treatment costs (costs in 2023 AUD). Abbreviations: Ab: antibody; Lab: laboratory; PoC: point-of-care; RNA: ribonucleic acid; Tx: treatment.

|  | **Average** | | | **Incremental to standard of care** | | |  |
| --- | --- | --- | --- | --- | --- | --- | --- |
| **Strategy** | **Cost per 1,000 screened** | **Completions per 1,000 screened** | **Cost-effectiveness ratio** | **Cost per 1,000 screened** | **Completions per 1,000 screened** | **Cost-effectiveness ratio** | **Willingness-to-pay per additional effect for which strategy maximizes net monetary benefit** |
| **Pharmaceutical Benefits Scheme ($36,111 per course)** | | | | | | | |
| Lab Ab / Lab RNA (re-collection) | $315,700 | 23 | $13,553 |  |  |  | $0 - $12,100 |
| Lab Ab / Lab RNA (reflex) | $436,800 | 33 | $13,127 | $121,100 | 10 | $12,132 | $12,200 - $20,900 |
| PoC Ab / PoC RNA | $579,000 | 40 | $14,448 | $263,300 | 17 | $15,691 | $21,000 - $66,300 |
| PoC RNA | $793,500 | 43 | $18,322 | $477,800 | 20 | $23,874 | $66,400 - $115,300 |
| PoC Ab / Lab RNA / early Tx | $1,283,200 | 48 | $26,986 | $967,500 | 24 | $39,885 | ≥$115,400 |
| PoC Ab / Lab RNA | $414,300 | 30 | $13,904 | $98,600 | 7 | $15,161 |  |
| Lab RNA | $483,600 | 33 | $14,534 | $167,900 | 10 | $16,823 |  |
| **Base-case ($13,495 per course)** | | | | | | | |
| Lab Ab / Lab RNA (re-collection) | $153,000 | 23 | $6,568 |  |  |  | $0 - $5,100 |
| Lab Ab / Lab RNA (reflex) | $204,400 | 33 | $6,141 | $51,400 | 10 | $5,147 | $5,200 - $11,000 |
| PoC Ab / PoC RNA | $279,600 | 40 | $6,976 | $126,600 | 17 | $7,544 | $11,100 - $37,000 |
| PoC Ab / Lab RNA / early Tx | $556,700 | 48 | $11,707 | $403,700 | 24 | $16,641 | ≥$37,100 |
| PoC Ab / Lab RNA | $206,200 | 30 | $6,919 | $53,200 | 7 | $8,176 |  |
| Lab RNA | $251,200 | 33 | $7,549 | $98,200 | 10 | $9,838 |  |
| PoC RNA | $399,900 | 43 | $9,235 | $247,000 | 20 | $12,340 |  |
| **Generic ($145 per course)** | | | | | | | |
| Lab Ab / Lab RNA (re-collection) | $56,900 | 23 | $2,444 |  |  |  | $0 - $1,000 |
| Lab Ab / Lab RNA (reflex) | $67,100 | 33 | $2,018 | $10,200 | 10 | $1,024 | $1,100 - $4,200 |
| PoC Ab / Lab RNA / early Tx | $127,600 | 48 | $2,683 | $70,700 | 24 | $2,913 | ≥$4,300 |
| PoC Ab / PoC RNA | $102,800 | 40 | $2,565 | $45,900 | 17 | $2,734 |  |
| PoC Ab / Lab RNA | $83,300 | 30 | $2,795 | $26,400 | 7 | $4,053 |  |
| Lab RNA | $114,000 | 33 | $3,425 | $57,000 | 10 | $5,714 |  |
| PoC RNA | $167,600 | 43 | $3,871 | $110,700 | 20 | $5,532 |  |

Supplementary figures


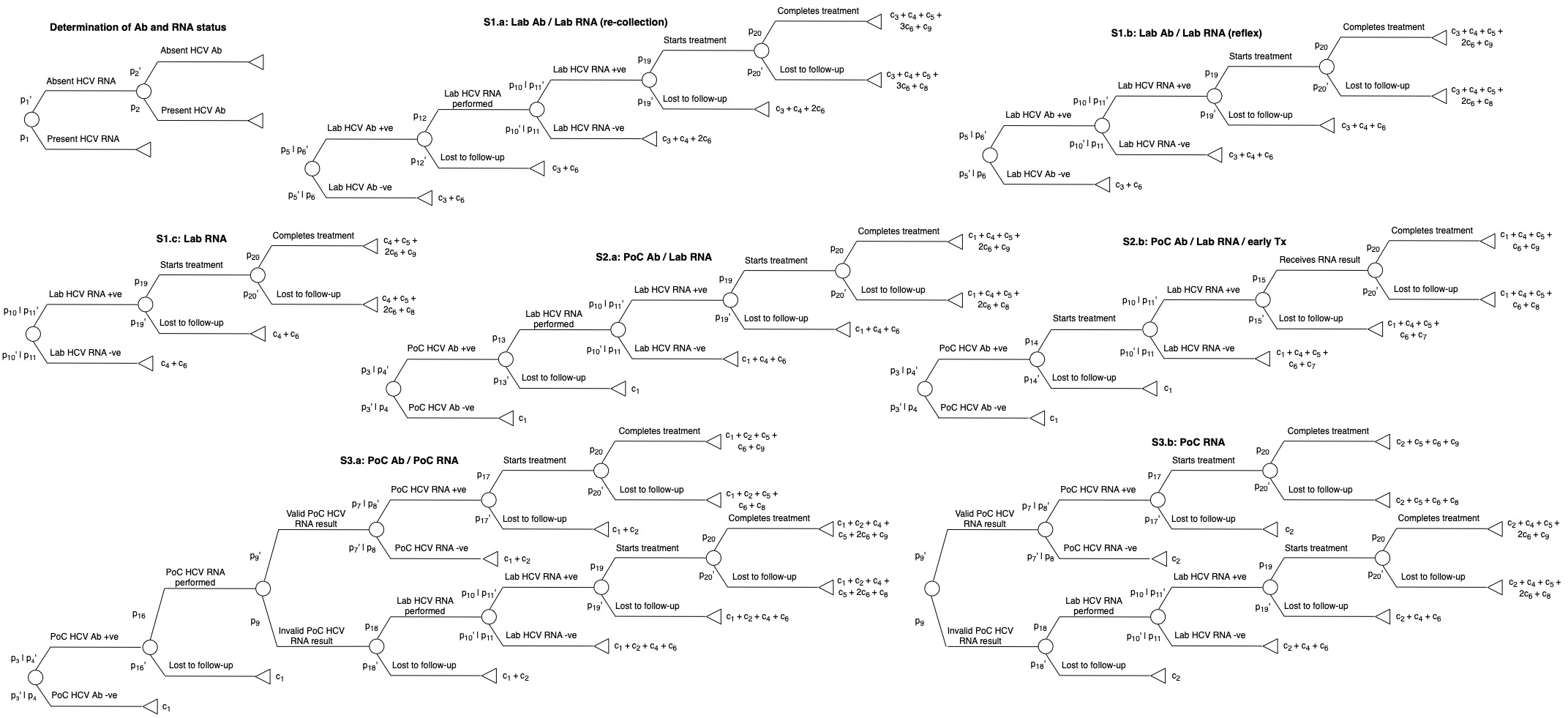


**Supplementary figure 1.** Modular structure of decision trees. Viraemia and antibody status are determined (top left), before cost and effect payoffs for each strategy. Branch labels refer to transition probabilities and terminal node labels to cost payoffs (Table 1). Health effect payoffs not shown. Abbreviations: Ab: antibody; HCV: hepatitis C virus; Lab: laboratory; PoC: point-of-care; RNA: ribonucleic acid; Tx: treatment.


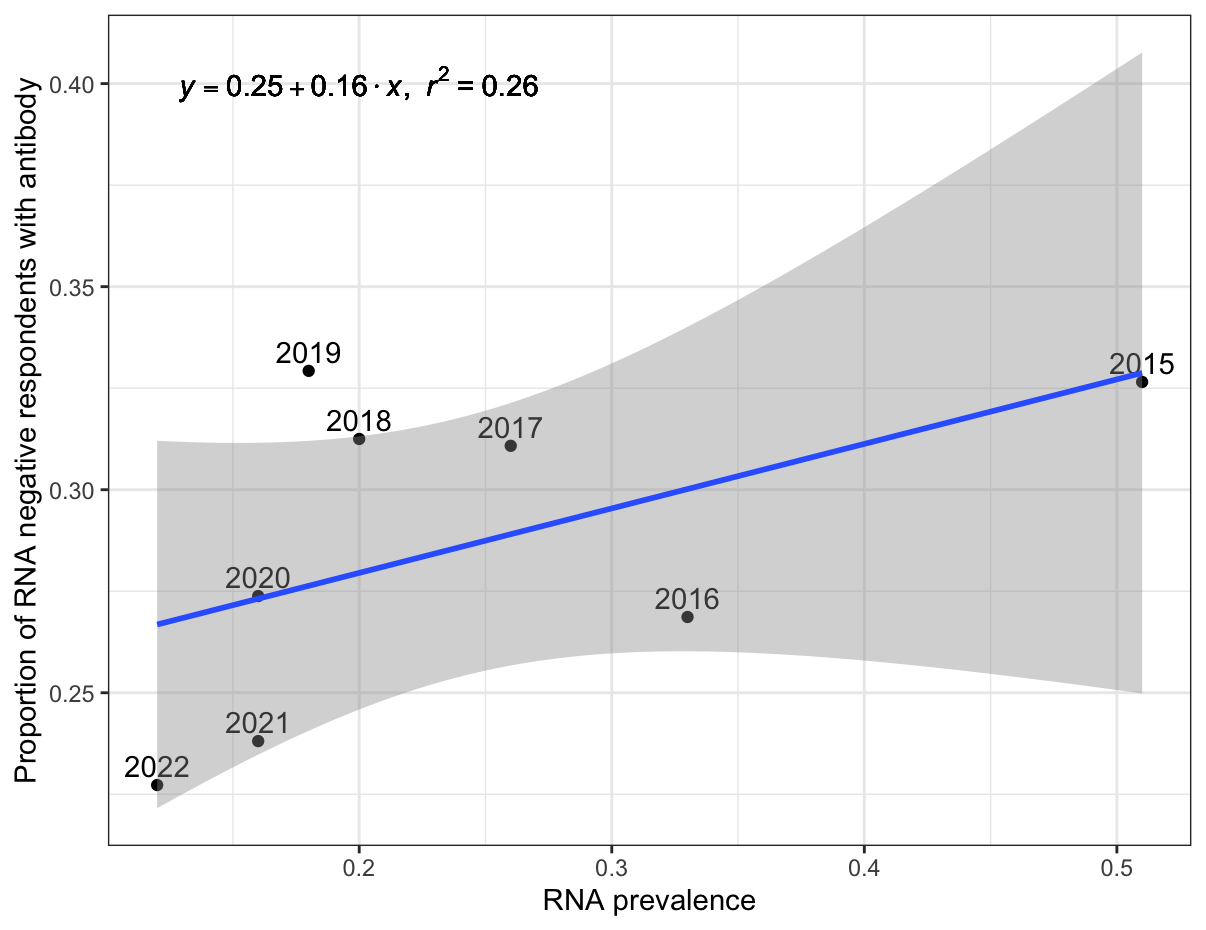


**Supplementary figure 2.** Correlation of RNA prevalence and proportion of HCV RNA negative respondents with detectable HCV antibody in Australian Needle and Syringe Program Surveys 2015-2022. Blue line and grey band show predictions and corresponding 95% confidence intervals for fitted linear model.


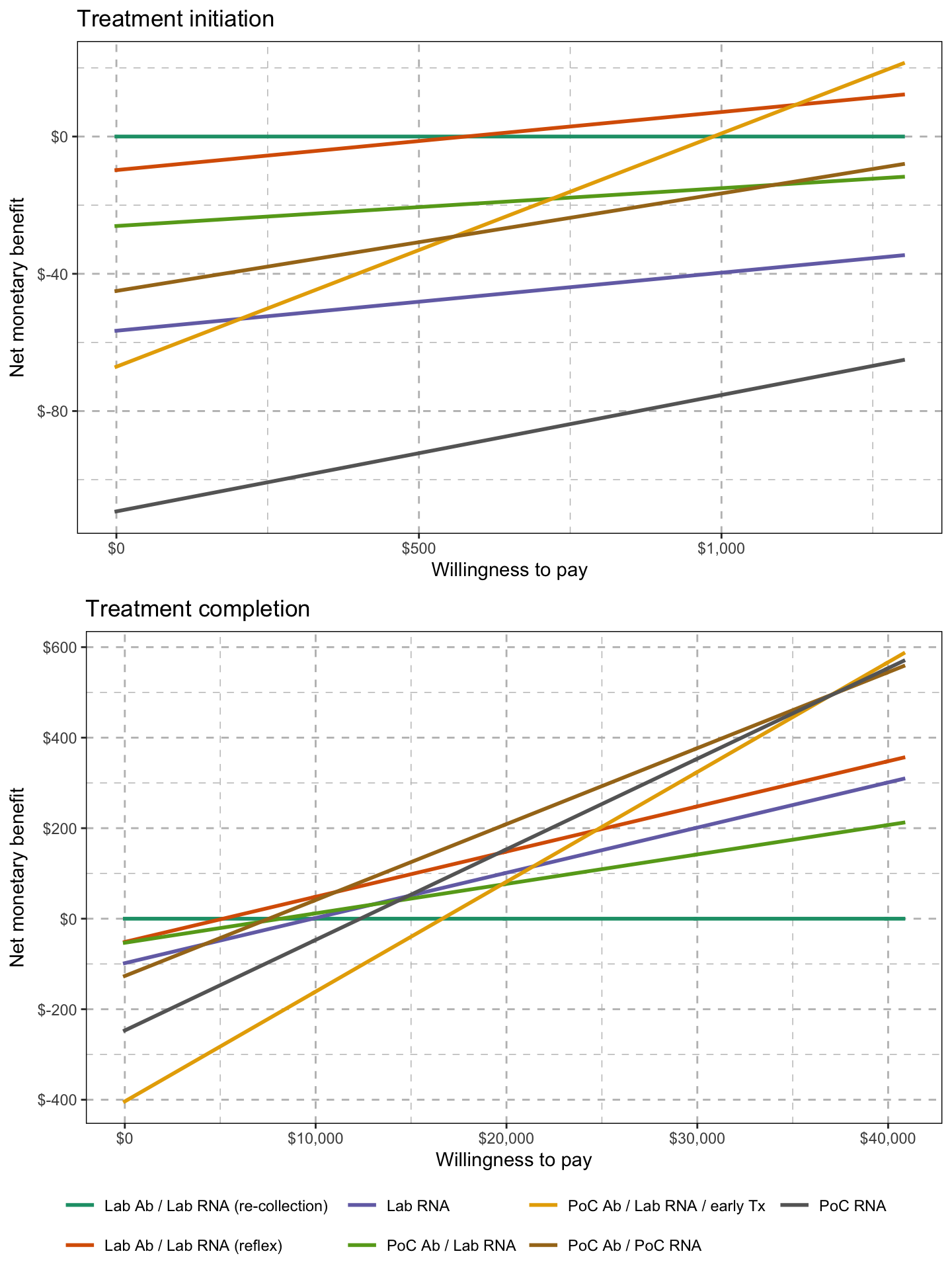


**Supplementary figure 3.** Estimated net monetary benefit (2023 AUD) of hepatitis C treatment initiation strategies by willingness to pay per additional treatment completion/initiation for treatment-naive people who inject drugs, under base-case assumptions. Abbreviations: Ab: antibody; Lab: laboratory; PoC: point-of-care; RNA: ribonucleic acid; Tx: treatment.


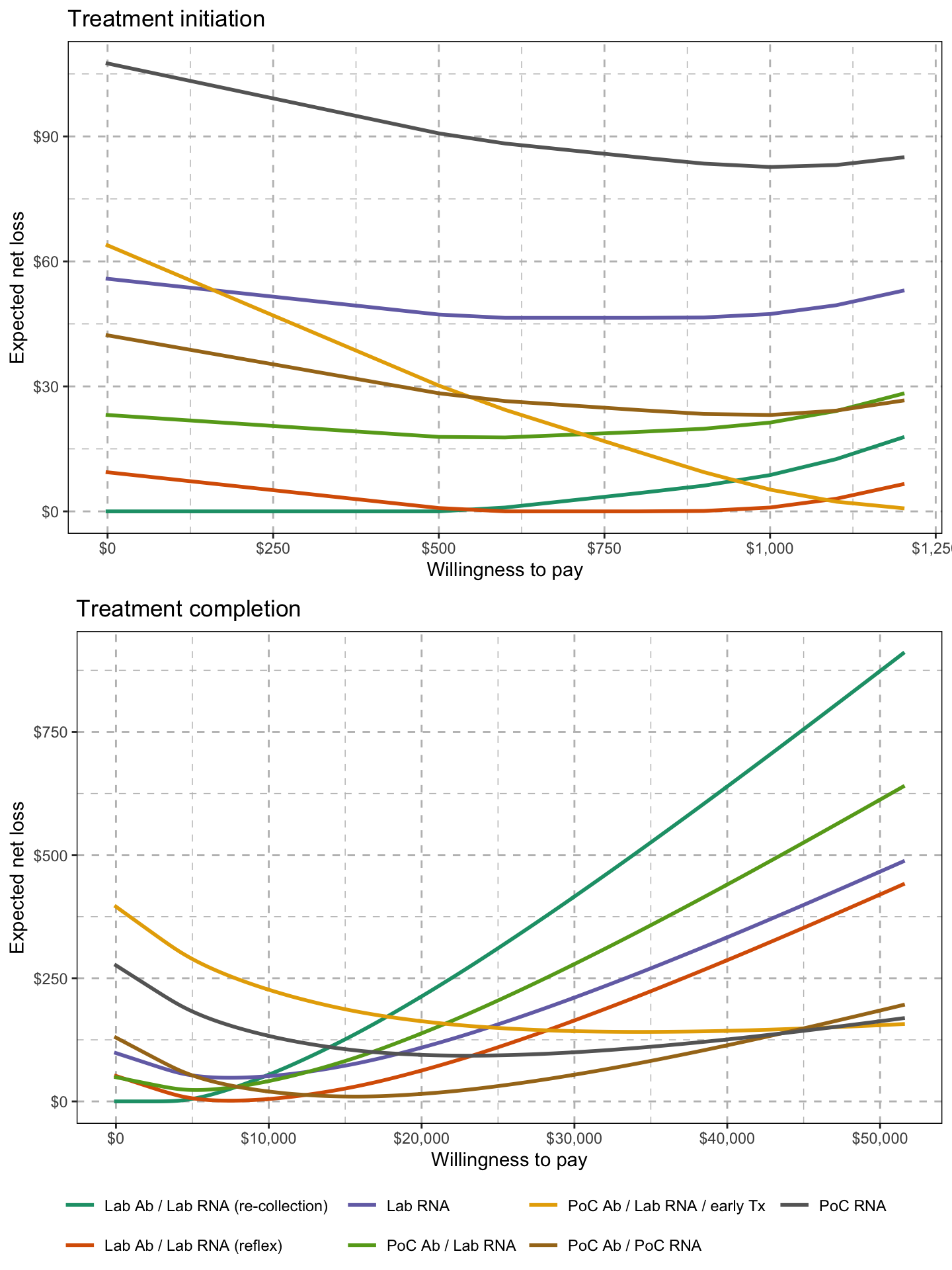


**Supplementary figure 4.** Expected net loss (loss resulting from choosing a strategy over the most cost-effective strategy, averaged over 1000 probabilistic iteration; 2023 AUD) of hepatitis C treatment initiation strategies by willingness to pay per additional treatment initiation/completion for treatment-naive people who inject drugs, under probabilistic assumptions. Abbreviations: Ab: antibody; Lab: laboratory; PoC: point-of-care; RNA: ribonucleic acid; Tx: treatment.


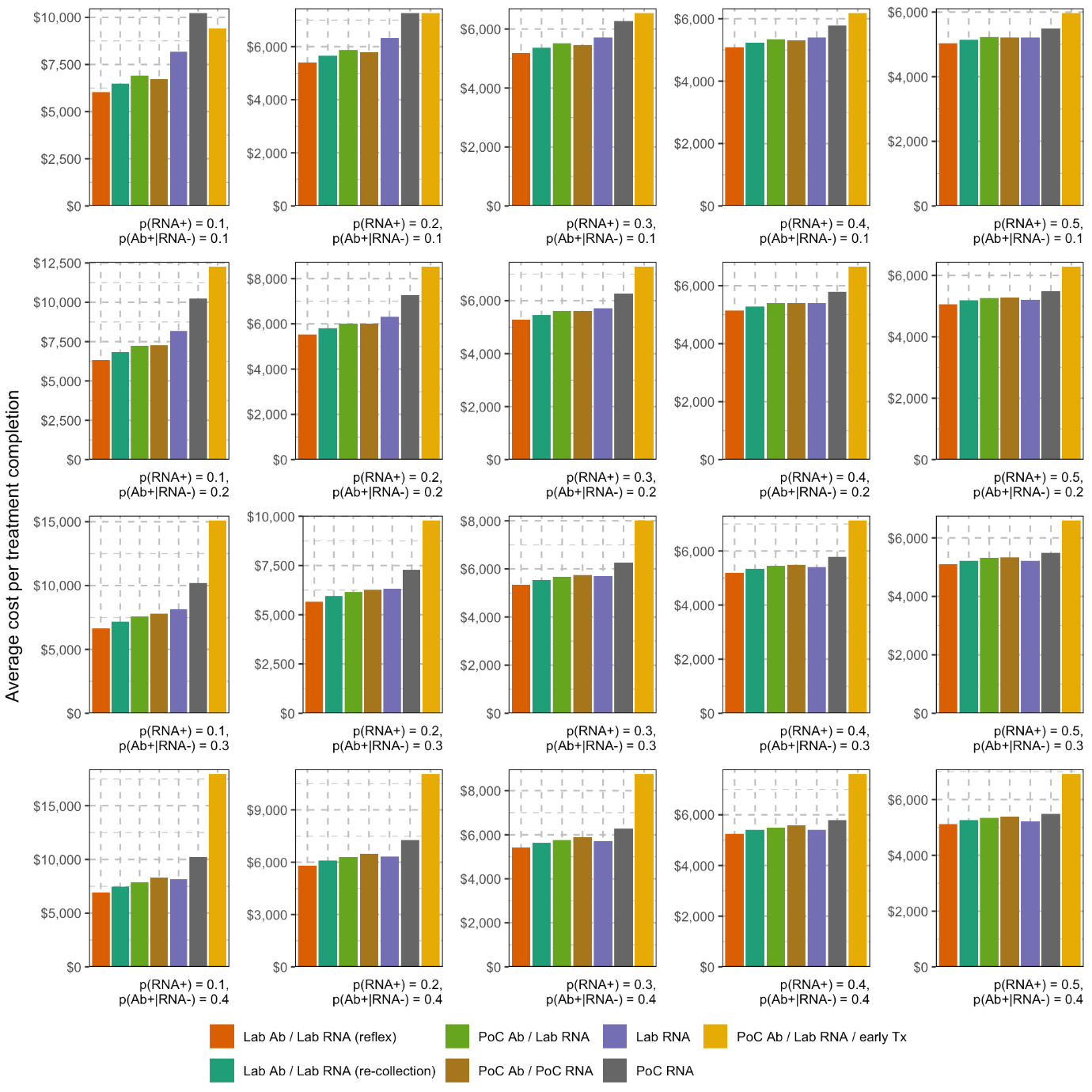


**Supplementary figure 5.** Estimated cost-effectiveness (2023 AUD) of hepatitis C treatment initiation strategies in achieving treatment completion for treatment-naive people who inject drugs, under varying prevalence (medium/high prevalence settings). Abbreviations: Ab: antibody; Lab: laboratory; PoC: point-of-care; RNA: ribonucleic acid; Tx: treatment.


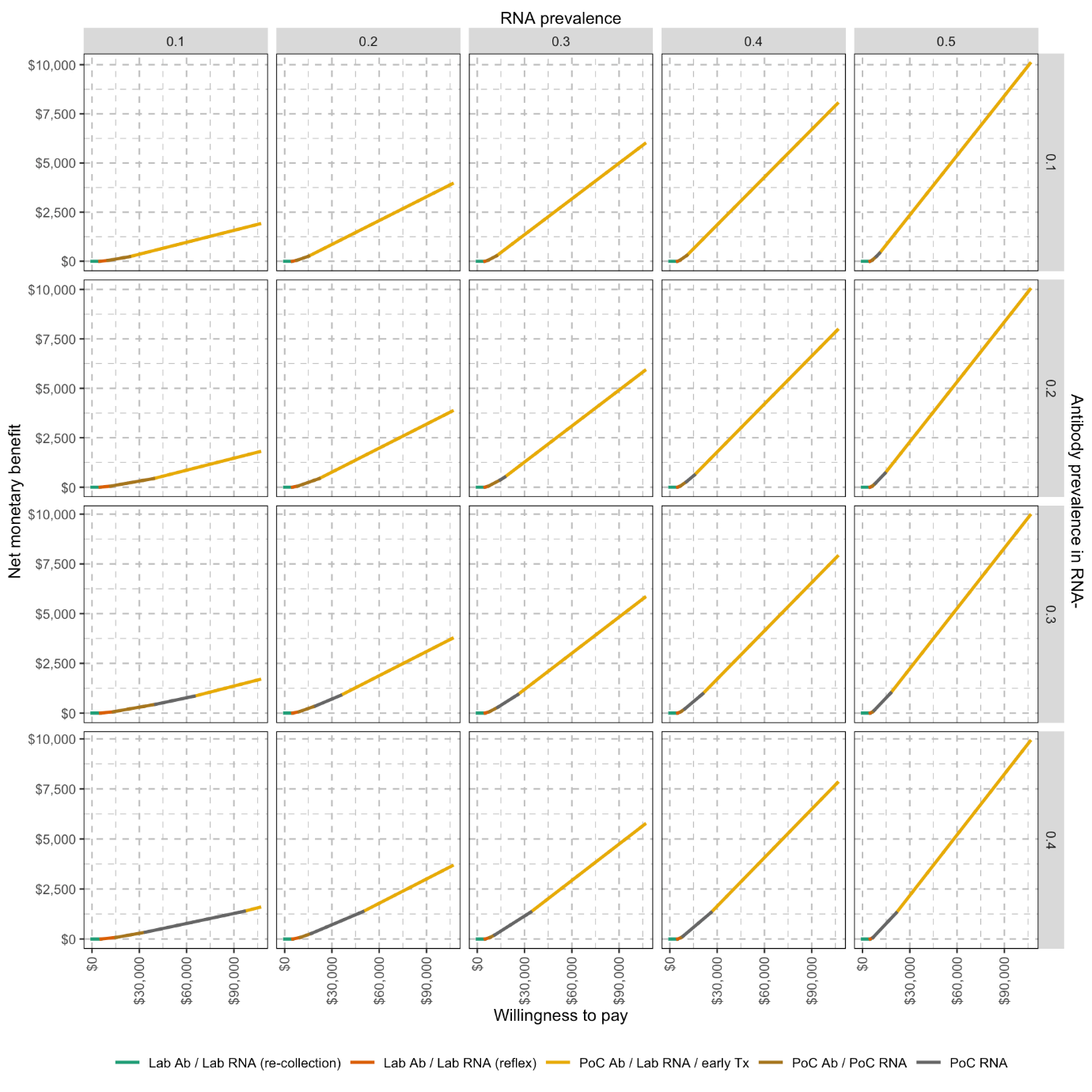


**Supplementary figure 6.** Estimated net monetary benefit (2023 AUD) of hepatitis C treatment initiation strategies by willingness to pay per additional treatment completion for treatment-naive people who inject drugs, under varying prevalence (medium/high prevalence settings). Only strategies on the net monetary benefit frontier are shown. Abbreviations: Ab: antibody; Lab: laboratory; PoC: point-of-care; RNA: ribonucleic acid; Tx: treatment.


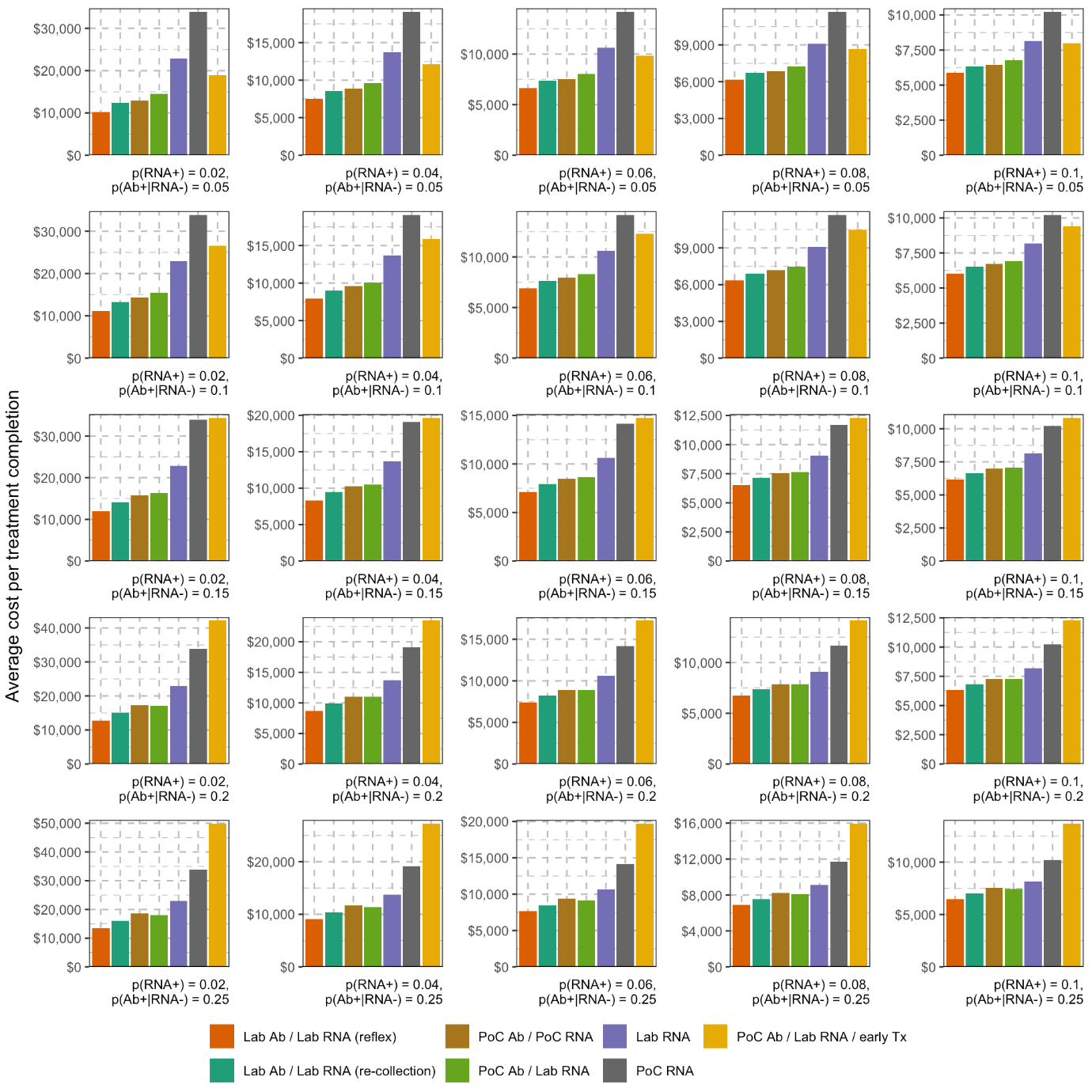


**Supplementary figure 7.** Estimated cost-effectiveness (2023 AUD) of hepatitis C treatment initiation strategies in achieving treatment completion for treatment-naive people who inject drugs, under varying prevalence (low prevalence settings). Abbreviations: Ab: antibody; Lab: laboratory; PoC: point-of-care; RNA: ribonucleic acid; Tx: treatment.


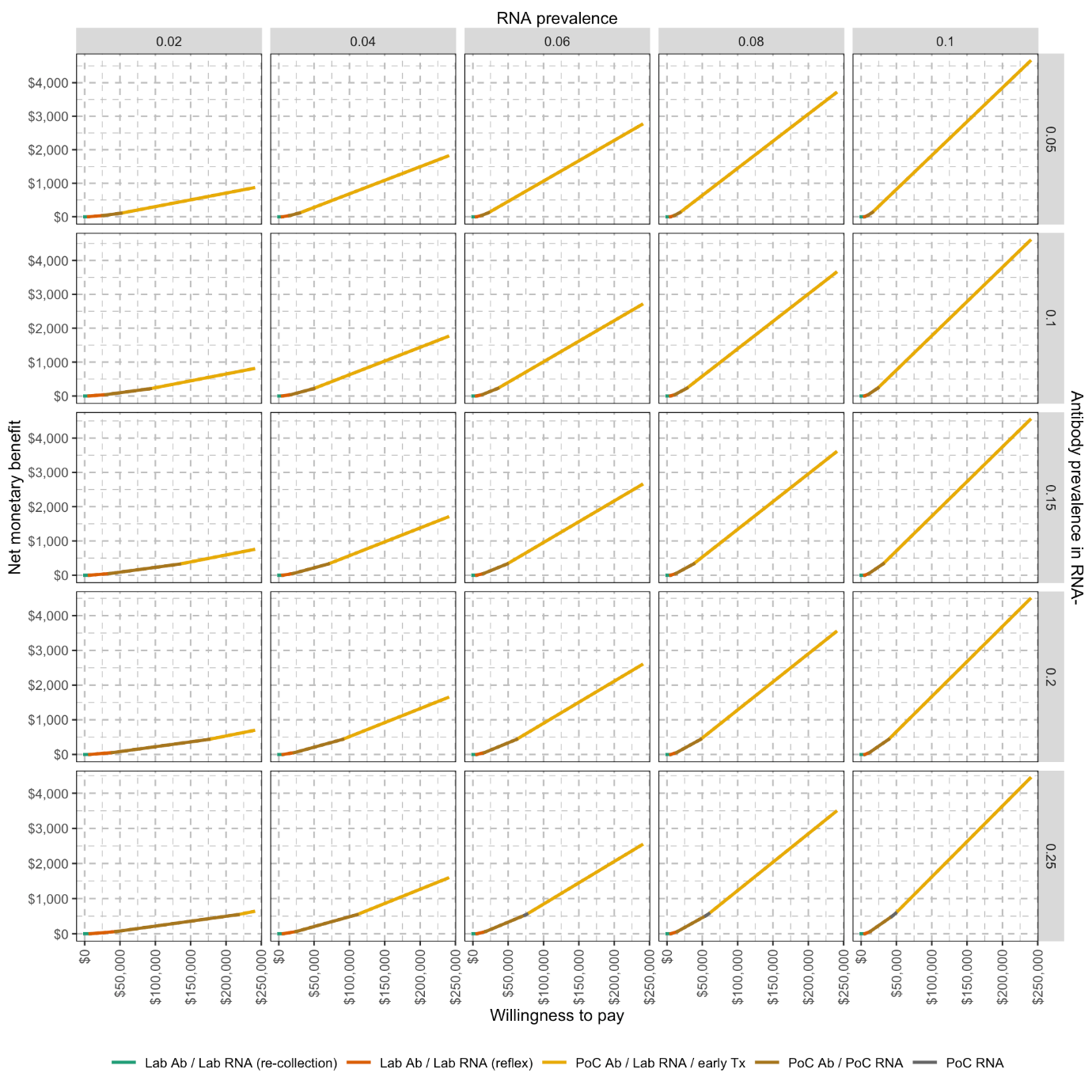


**Supplementary figure 8.** Estimated net monetary benefit (2023 AUD) of hepatitis C treatment initiation strategies by willingness to pay per additional treatment initiation for treatment-naive people who inject drugs, under varying prevalence (low prevalence settings). Only strategies on the net monetary benefit frontier are shown. Abbreviations: Ab: antibody; Lab: laboratory; PoC: point-of-care; RNA: ribonucleic acid; Tx: treatment.


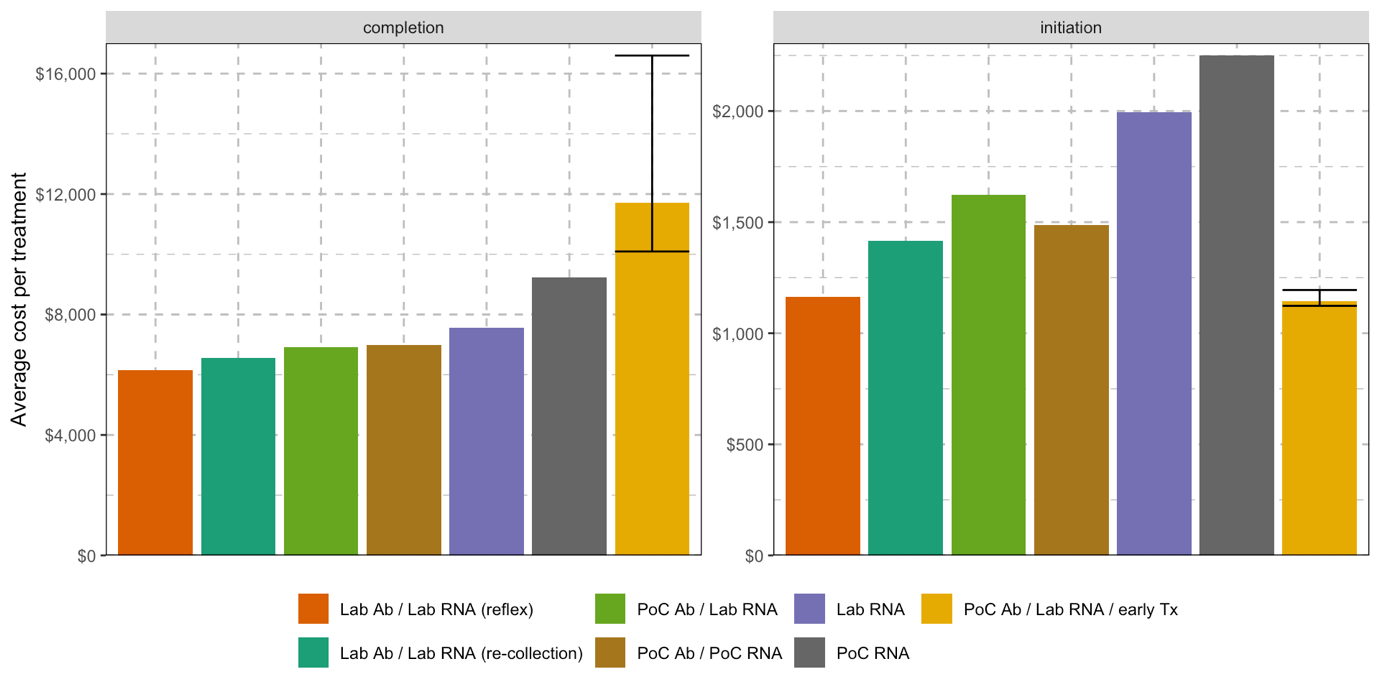


**Supplementary figure 9.** Estimated cost-effectiveness (2023 AUD) of a point-of-care antibody/laboratory RNA/early treatment hepatitis C treatment initiation strategy in achieving treatment completion/initiation for treatment-naive people who inject drugs under optimistic and pessimistic loss to follow up assumptions (error bars). Estimates for other strategies under base-case assumptions shown for comparison. Abbreviations: Ab: antibody; Lab: laboratory; PoC: point-of-care; RNA: ribonucleic acid; Tx: treatment.


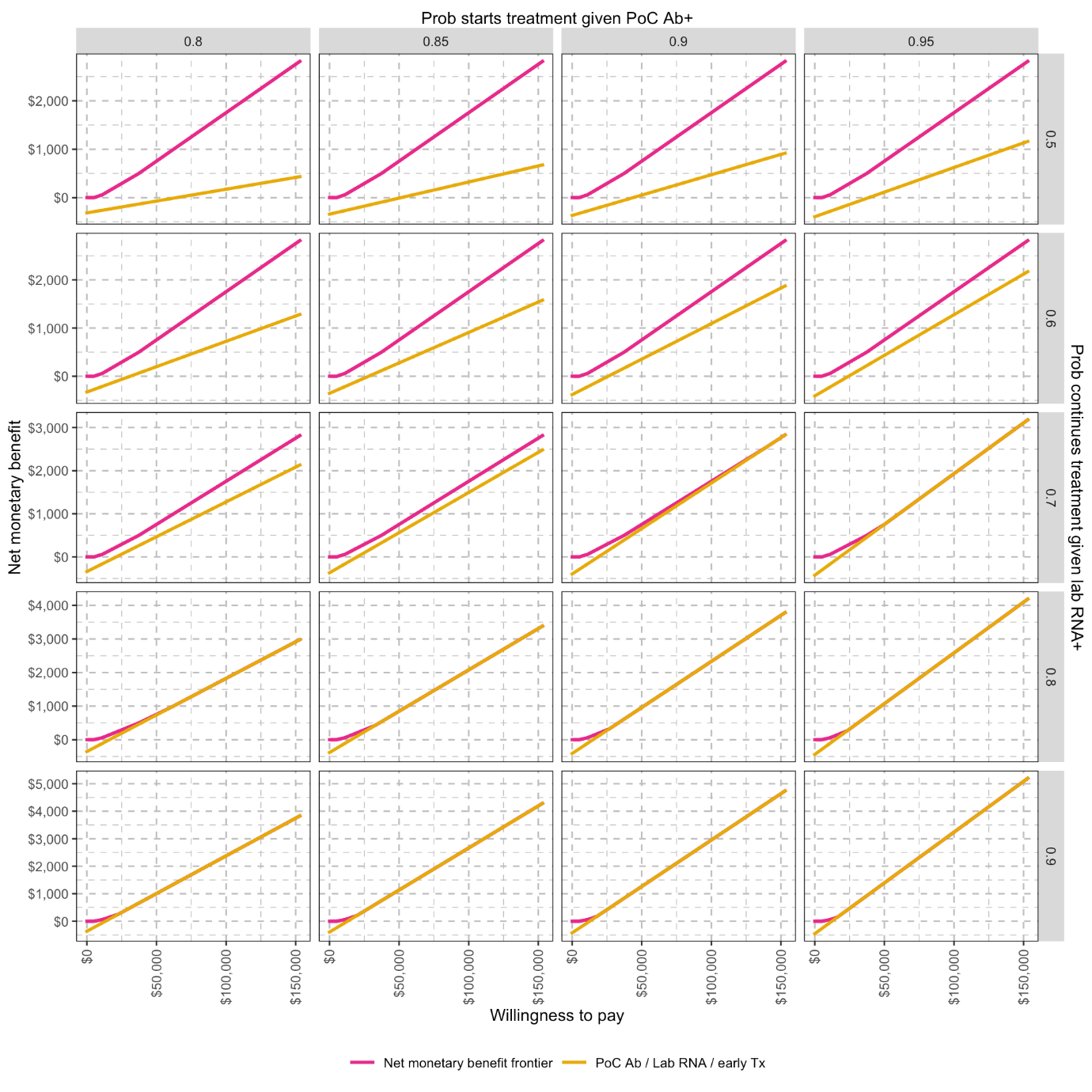


**Supplementary figure 10.** Estimated net monetary benefit (2023 AUD) of a point-of-care antibody/laboratory RNA/early treatment hepatitis C treatment initiation strategy under varying loss to follow up assumptions, and the net monetary benefit frontier presented by competing strategies, by willingness to pay per additional treatment completion for treatment-naive people who inject drugs. Abbreviations: Ab: antibody; Lab: laboratory; PoC: point-of-care; RNA: ribonucleic acid; Tx: treatment.


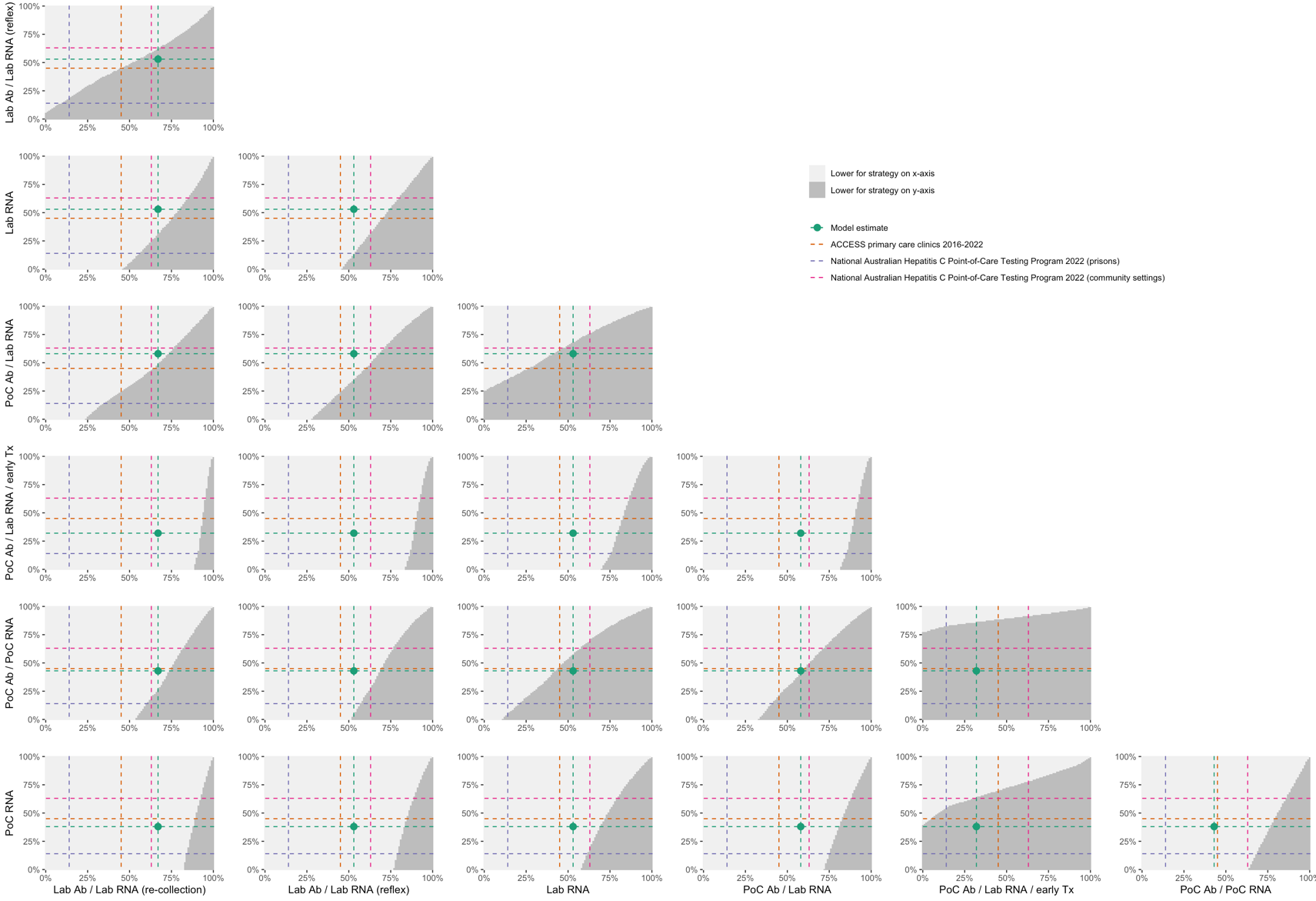


**Supplementary figure 11.** Effect of varying loss to follow-up prior to RNA-confirmed treatment initiation (axes) on relative cost per treatment completion of hepatitis C treatment initiation strategies for treatment-naive people who inject drugs. Dashed lines show assumed loss-to-follow up in the base-case and reported values in Australian Collaboration for Coordinated Enhanced Sentinel Surveillance of Sexually Transmissible Infections and Blood Borne Viruses (ACCESS) primary care clinics (1) and National Australian Hepatitis C Point-of-Care Testing Program (2) for comparison. Abbreviations: Ab: antibody; Lab: laboratory; PoC: point-of-care; RNA: ribonucleic acid; Tx: treatment.


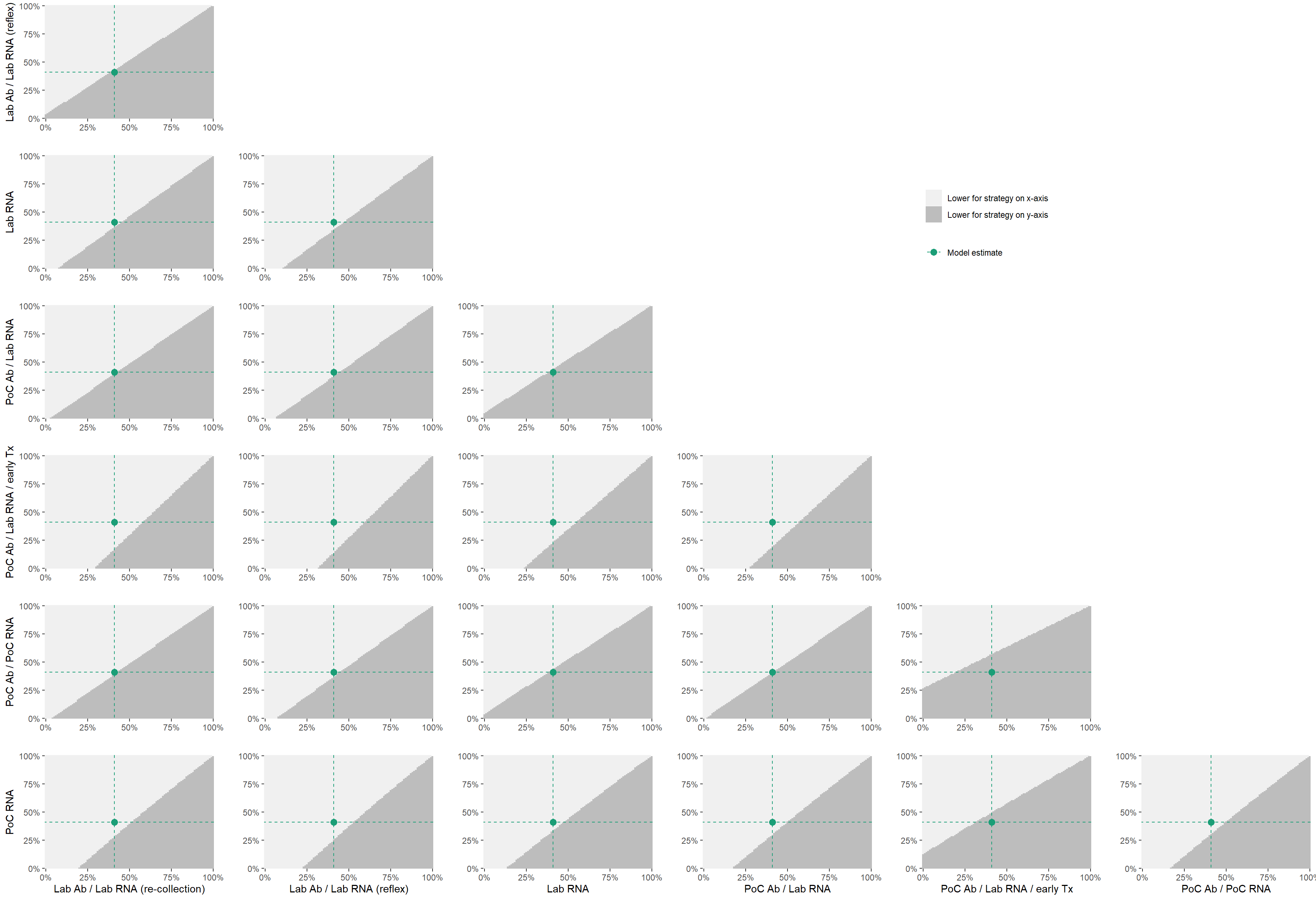


**Supplementary figure 12.** Effect of varying loss to follow-up after RNA-confirmed treatment initiation (axes) on relative cost per treatment completion of hepatitis C treatment initiation strategies for treatment-naive people who inject drugs. Dashed lines show assumed loss-to-follow up in the base-case. Abbreviations: Ab: antibody; Lab: laboratory; PoC: point-of-care; RNA: ribonucleic acid; Tx: treatment.

**References:**

1. Burnet Institute and Kirby Institute. Australia’s progress towards hepatitis C elimination: annual report 2023. Melbourne: Burnet Institute; 2023.

2. Grebely J, Markus C, Causer LM, Silk D, Comben S, Lloyd AR, et al. A national programme to scale-up decentralised hepatitis C point-of-care testing and treatment in Australia. The Lancet Gastroenterology & Hepatology. 2023;8(3):204-7.

**Alt text for supplementary figures:**

**Supplementary figure 1:** Decision trees for the seven strategies evaluated.

**Supplementary figure 2:** Scatter plot showing correlation between RNA prevalence and proportion of HCV RNA negative respondents with detectable HCV antibody in Australian Needle and Syringe Program Surveys. There is a rough positive linear relationship with Pearson correlation coefficient of 0.26.

**Supplementary figure 3:** Net monetary benefit curves for treatment initiation and completion for the seven strategies evaluated.

**Supplementary figure 4:** Expected net loss curves for treatment initiation and completion for the seven strategies evaluated.

**Supplementary figure 5:** Column charts showing estimated average cost per treatment completion under alternative high prevalence assumptions.

**Supplementary figure 6:** Net monetary benefit curves for treatment completion under alternative high prevalence assumptions

**Supplementary figure 7:** Column charts showing estimated average cost per treatment completion under alternative low prevalence assumptions.

**Supplementary figure 8:** Net monetary benefit curves for treatment completion under alternative low prevalence assumptions.

**Supplementary figure 9:** Column charts showing estimated average cost per treatment initiation and completion for the immediate treatment strategy under optimistic and pessimistic follow-up assumptions.

**Supplementary figure 10:** Net monetary benefit curves for treatment completion under alternative follow up assumptions for the immediate treatment strategy.

**Supplementary figure 11:** Pairwise comparisons of strategies evaluated under alternative follow up assumptions, shaded to show the combination of assumed loss-to-follow-up prior to treatment initiation under which either strategy has lower cost per treatment completion.

**Supplementary figure 12:** Pairwise comparisons of strategies evaluated under alternative follow up assumptions, shaded to show the combination of assumed loss-to-follow-up after treatment initiation under which either strategy has lower cost per treatment completion.
